# Microenvironment Matters: Copper–Carbon Composites Enable a Highly Efficient Carbon Dioxide Reduction Reaction to C_2_ Products

**DOI:** 10.1021/acsami.4c20586

**Published:** 2025-02-04

**Authors:** Yu-Jhih Shen, Yung-Hsi Hsu, Yu-Chia Chang, Jian-Jie Ma, Kang-Shun Peng, Ying-Rui Lu, Shao-Hui Hsu, Sung-Fu Hung

**Affiliations:** † Department of Applied Chemistry and Center for Emergent Functional Matter Science, National Yang Ming Chiao Tung University, Hsinchu 300, Taiwan; ‡ Department of Medicinal and Applied Chemistry, Kaohsiung Medical University, Kaohsiung 807, Taiwan; § National Synchrotron Radiation Research Center, Hsinchu 300, Taiwan; ∥ Taiwan Semiconductor Research Institute, National Applied Research Laboratories, Hsinchu 300, Taiwan

**Keywords:** CO_2_RR, metal organic framework, MOF-derived catalyst, X-ray absorption spectroscopy, in situ.

## Abstract

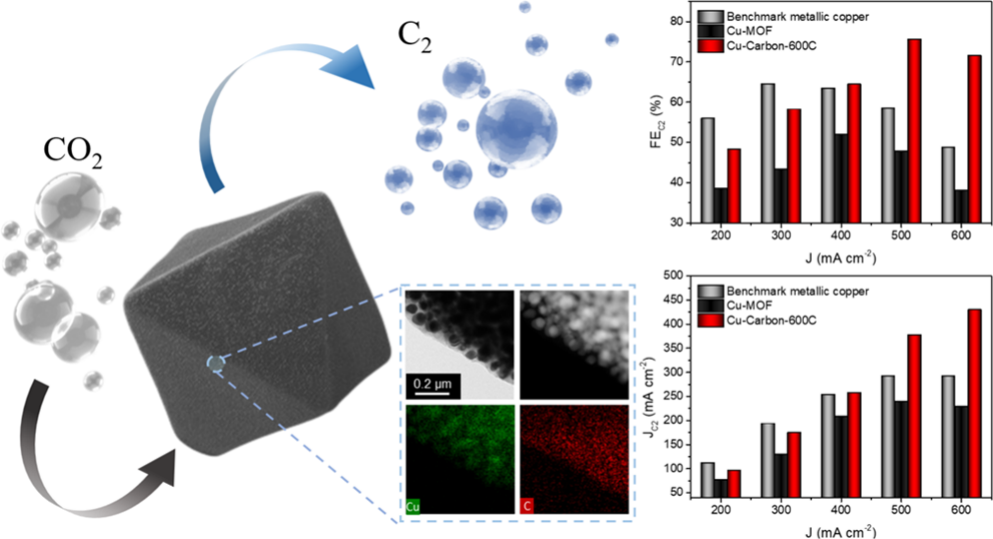

Copper is the catalyst widely used to produce multicarbon products for the carbon dioxide reduction reaction (CO_2_RR). The surrounding microenvironment of copper plays a crucial role in determining its catalytic activity and selectivity. In this study, we compare three copper electrocatalysts with different microenvironments: pure metallic copper, a copper metal–organic framework (MOF), and a MOF-derived copper–carbon composite. *Operando* X-ray absorption spectroscopy, transmission electron microscopy, and Raman spectroscopy reveal that copper in the copper–carbon composite remains in a metallic state, encapsulated by a carbon matrix. The composite catalyst achieves a Faradaic efficiency of 75.6% for C_2_ products, including ethylene and ethanol, at a current density of 500 mA cm^–2^, with a C_2_ current density of 377.9 mA cm^–2^. This performance suppresses pure metallic copper, which reaches an optimal Faradaic efficiency of 64.5% for C_2_ products at a current density of 300 mA cm^–2^, with a C_2_ current density of 193.5 mA cm^–2^. The copper–carbon composite also significantly overperforms the copper-MOF catalyst, which shows an optimal Faradaic efficiency of 52.0% for C_2_ products at a current density of 400 mA cm^–2^, with a C_2_ current density of 208.0 mA cm^–2^. These findings highlight the importance of the microenvironment near active copper sites in determining CO_2_RR efficiency. We hope that our results provide valuable insights for advancing catalyst design in carbon dioxide reduction, contributing to reduced carbon emissions and improved environmental sustainability.

## 1 Introduction

One of the greatest challenges of our era is addressing the growing global energy demand in a sustainable and clean manner. While fossil fuels have significantly improved our quality of life, their extraction and combustion have led to serious environmental issues, including pollution and high carbon emissions that contribute to global warming. To effectively address these complex environmental problems, it is crucial to transition the energy landscape from fossil fuels to low-carbon, and eventually carbon-free, technologies. Among these, the electrochemical CO_2_ reduction reaction (CO_2_RR) stands out as a highly promising approach, offering the ability to close the carbon cycle by converting CO_2_ into valuable products with high energy density and adjustable product selectivity.^1−3^

Various transition metal-based materials have been investigated as electrocatalysts for CO_2_RR. Among these, copper-based materials have emerged as particular promise due to their ability to convert CO_2_ into valuable multicarbon products such as ethylene and ethanol, which hold significant market value and potential for large-scale production.^4−6^ However, several challenges hinder their practical application, including low selectivity and partial current densities, and competition from the hydrogen evolution reaction (HER).^7−10^ Improving the activity and selectivity of copper-based catalysts for producing multicarbon products through CO_2_RR can be achieved by tuning the adsorption energy of the intermediates,^11^ regulating the valence states of copper active sites,^12−14^ and adapting the microenvironments near active copper sites.^15−17^

Copper-based electrocatalysts with varying microenvironments have demonstrated distinct selectivity for CO_2_RR. For instance, when active sites of metallic copper are surrounded by carbon black, methane is predominantly produced;^18,19^ when capped by nitrogen doped carbon, ethanol becomes the main product;^20^ when decorated with carbon nanotubes, the CO_2_RR primarily yields ethylene.^21^ These findings highlight the significant influence that microenvironments have on both the selectivity and activity of copper-based catalysts.

Carbon-supported catalysts have garnered significant attention in various applications due to their low cost and high catalytic activity.^22−26^ And MOF-derived carbon-based materials become a new approach to carbon support catalysts which offer several significant advantages that make them highly effective in catalytic applications. Their high porosity, adjustable pore size, and large specific surface area facilitate the adsorption of molecules near the reaction substrate, enhance material transport, and activate catalytic centers.^27,28^ Additionally, the metal nodes and organic linkers in MOFs contribute to catalyzing complex reactions through charge transfer interactions with active centers, such as coordination or π–π forces, thereby improving the chemical microenvironment within the pore structures.^29−32^ Unlike traditional carbon-based catalysts, the MOF shells effectively encapsulate the active centers, preventing the leaching or agglomeration of metal sites and ensuring excellent stability during catalytic processes.^33,34^ Furthermore, the periodic arrangement of transition metal units within MOFs ensures an even dispersion of active sites, which is critical for consistent catalytic performance.^35^ By tailoring synthesis strategies, MOF-derived carbon-based materials can selectively confine metals of various sizes, making them increasingly popular for use in a wide range of electrocatalytic reactions.^36,37^

Herein, in comparison to the benchmark pure metallic copper catalyst (showing an optimal Faradaic efficiency of 64.5% for C_2_ products at a current density of 300 mA cm^–2^, with a C_2_ current density of 193.5 mA cm^–2^), the metallic copper with a distinct microenvironment–a copper–carbon composite derived from a copper metal–organic framework–achieved a significantly higher Faradaic efficiency of 75.6% for C_2_ products at a current density of 500 mA cm^–2^, with a C_2_ current density of 377.9 mA cm^–2^. Additionally, the composite overperformed the copper-organic framework, which reached only an optimal Faradaic efficiency of 52.0% for C_2_ products at a current density of 400 mA cm^–2^, with a C_2_ current density of 208.0 mA cm^–2^. These results affirm that the microenvironment surrounding active copper sites plays a critical role in determining CO_2_RR performance, contributing to atmospheric carbon dioxide mitigation and supporting efforts toward carbon neutrality.

## 2 Experimental Section

### 2.1 Chemicals

Cu(NO_3_)_2_·3H_2_O (99%) was purchased from ACROS. 1,3,5-benzene tricarboxylic acid (98%) was purchased from Thermo Scientific and Nafion solution (5% in lower aliphatic alcohols and water, contains 15–20% water). All chemicals were used without further purification.

### 2.2 Preparation of Cu-MOF (Copper(II)-benzene-1,3,5-tricarboxylate)

The method for preparing the Cu-MOF was modified in accordance with a reported work in the preparation.^38^ 2.952 g Cu(NO_3_)_2_·3H_2_O was dissolved in 40 mL of a mixture solvent with ethanol/DI water volumetric ratio of 1:1. 1.713 g of 1,3,5-benzene tricarboxylic acid was dissolved in 40 mL of a mixture solvent with ethanol/DI water volumetric ratio of 1:1. Then the Cu(NO_3_)_2_·3H_2_O solution and 1,3,5-benzene tricarboxylic acid solution were transfer into a 100 mL Teflon-lined stainless-steel autoclave and heated at 120 °C for 24 h. After cooling the precipitation was retrieved and washed with ethanol three times then dried in vacuum at 80 °C overnight to obtain Cu-MOF.

### 2.3 Preparation of Cu-Graphene-XC

Cu-MOF was pyrolyzed at 400, 500, 600 °C in flowing N_2_ (20 sccm) and 700,800 °C in flowing Ar (20 sccm) for 2h to obtain Cu-Graphene-XC (X indicates the pyrolysis temperature).

### 2.4 Preparation of the Gas-Diffusion Electrode

One mg of catalyst was dispersed in 200 μL of methanol with 4 μL of Nafion solution (∼5 wt %). The dispersed ink was uniformly spray coated on PTFE substrate with a loading amount of 1 mg/cm^2^.

### 2.5 Preparation of the Benchmark Metallic Copper Gas-Diffusion Electrode

For a pure metallic copper *gas-diffusion electrode*, a 300 nm of copper was sputtered onto a PTFE substrate via magnetron sputtering, using a pure Cu target (99.99%) under an Ar flow rate of 3 mTorr and a DC power of 30 W. The base pressure for the sputtering process was maintained below 5 × 10^–6^ Torr, acquired through the use of a turbomolecular pump.

### 2.6 Characterization

The microstructure was examined using a field-emission scanning electron microscope (FESEM, JEOL, JSM-6700F) equipped with energy-dispersive X-ray spectroscopy (EDX, Oxford Instrument INCAx-sight 7557). Lattice fringes and elemental mapping for ex-situ measurements were obtained using a field-emission transmission electron microscope (FE-TEM, JEOL-2100F) equipped with EDX (Oxford Instrument XMaxN TSR) at the Department of Chemistry, National Taiwan University, Taiwan. The BET surface area was determined using nitrogen gas adsorption data within a relative pressure (P/P_0_) range of 0.002 to 0.05. The measurements were performed using a Micromeritics 3Flex instrument equipped with the Smart VacPrep 067 system. Crystal structures were determined by synchrotron X-ray diffraction analysis using an incident X-ray wavelength of 0.7749 Å at the BL01C2 beamline of TLS, NSRRC. X-ray photoelectron spectroscopy (XPS) measurements were carried out on an ULVAC-PHI PHI 5000 Versaprobe II spectrometer under a pressure of 2.4 × 10^–10^ torr, using a monochromatic Al K_α_ X-ray source (1486.60 eV). The binding energies (BEs) of the elements were calibrated to adventitious carbon at 284.6 eV. The inductively coupled plasma Mass spectrometry (ICP-MS) test was examined by Thermo Fisher Scientific iCAP TQ. X-ray absorption spectroscopy (XAS), including X-ray absorption near-edge spectra (XANES) and extended X-ray absorption fine structure (EXAFS) of the Cu K-edge, was collected in fluorescence mode at the BL32A beamline of TPS, NSRRC. The pre-edge baseline was subtracted, and the spectra were normalized to the postedge. EXAFS analysis involved applying a Fourier transform to the k^2^-weighted EXAFS oscillations to evaluate the contribution of each bond pair to the Fourier transform peaks.

### 2.7 Electrochemical Measurement

The electrochemical properties were examined using a Biologic VSP-3e potentiostat in a flow cell reactor, where a gas diffusion electrode (GDE) served as the working electrode (WE), nickel foam as the counter electrode (CE), and a saturated Ag/AgCl electrode as the reference electrode (RE). The WE and CE were separated by an anion exchange membrane. Cu-MOF utilized a 1.0 M KHCO_3_ aqueous solution as the electrolyte to preserve the integrity of the MOF structure and prevent damage that could occur in an alkaline environment. In contrast, the other samples employed a 1.0 M KOH aqueous solution as the electrolyte. CO_2_ was supplied at a flow rate of 50 sccm. The potentials were converted to values relative to the reversible hydrogen electrode (RHE) using the equation: *E*_RHE_ = *E*_Ag/AgCl_ + 0.0591 × pH + *E*_Ag/AgCl_^0^, where *E*_Ag/AgCl_^0^ (0.210 V) is the standard potential of Ag/AgCl relative to the standard hydrogen electrode (SHE) at 25 °C, and via *iR* compensation. The gaseous products were analyzed using gas chromatography (Agilent 8860) equipped with a thermal conductivity detector and a flame ionization detector. The liquid products were analyzed using high performance liquid chromatography (Agilent 1260 Infinity II) equipped with a variable wavelength detector and a refractive index detector. The isotope analysis was analyzed using gas chromatography (Agilent 5975C) equipped with a mass spectrum (Agilent 5975C).

### 2.8 Operando XAS Measurement

#### 2.8.1 Operando

XAS was performed under the same conditions as the electrochemical tests, using a custom-designed flow cell with a Kapton tape-sealed opening in the gas chamber. X-ray absorption spectroscopy signals were collected in total fluorescence yield mode at BL32A beamline of TPS, NSRRC.

### 2.9 Raman and Operando Raman Measurement

#### 2.9.1 Raman and Operando

Raman was performed under the same conditions as the electrochemical tests, using a custom-designed flow cell and 785 nm diode laser as incident light. Raman signals were collected by Renishaw inVia.

## 3 Results and Discussion

**Figure 1:**
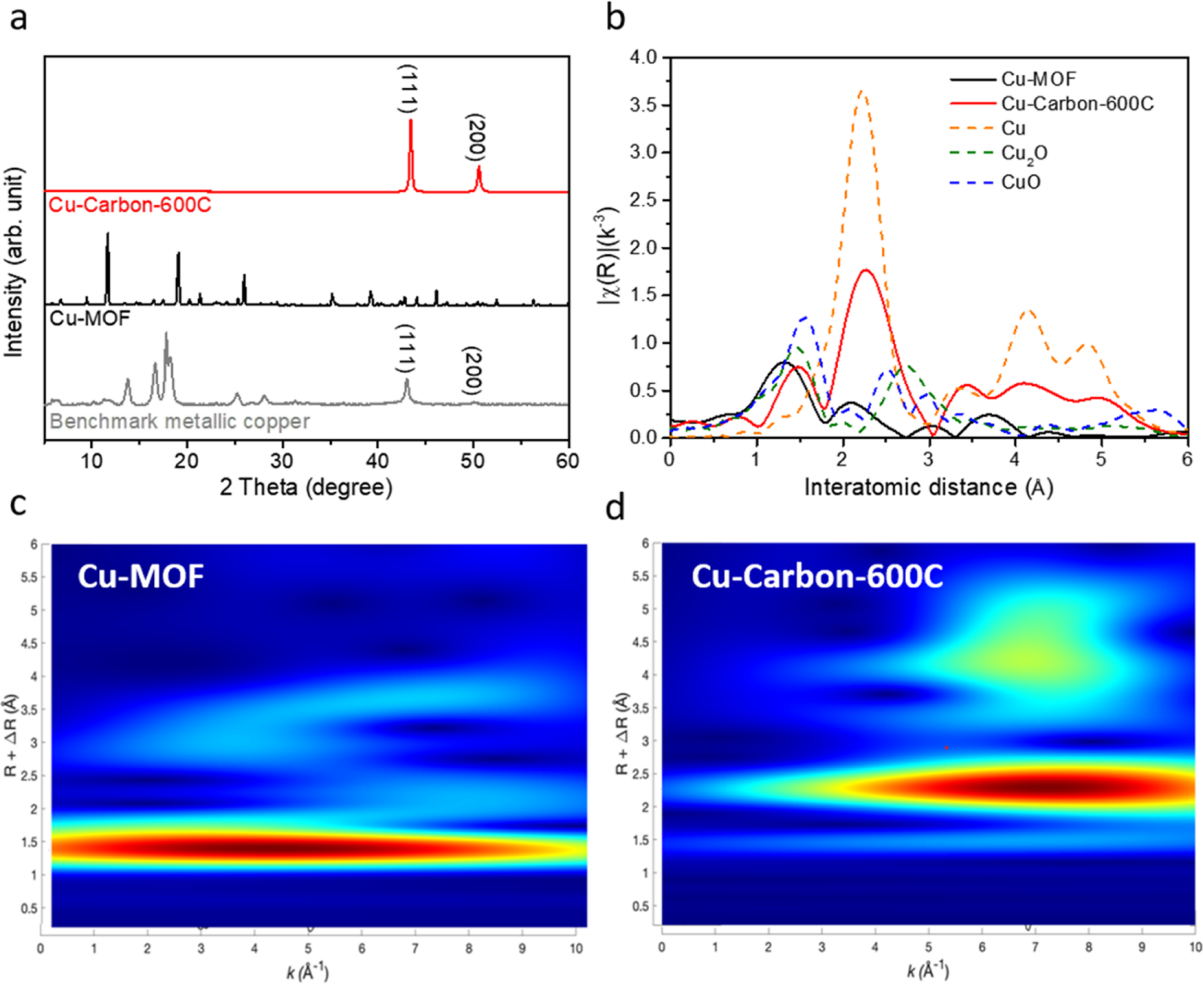
(a) XRD of benchmark metallic Cu, Cu-MOF, and Cu-Carbon-600C. (b) EXAFS of Cu-MOF and Cu-Carbon-600C. Wavelet analysis of (c) Cu-MOF and (d) Cu-Carbon-600C.

To identify the microenvironment surrounding the active copper sites and the crystal structure, we used synchrotron X-ray diffraction (XRD) to analyze Cu-Carbon, Cu-MOF, and the benchmark metallic copper. As shown in Figures 1a and S1, the XRD characteristic peaks of Cu-MOF correspond to the standard spectrum (JCPDS No. 00-062-1183).^39^ The samples subjected to heat treatment exhibited significant structural changes compared to the original Cu-MOF; the characteristic peaks of Cu-MOF disappeared and were replaced by a prominent peak at 43.4°, corresponding to the (111) facet of metallic copper, indicating the transformation of copper into metallic copper in the pyrolyzed samples. The peak corresponding to metallic copper was also observed in the benchmark metallic copper sample, along with additional peaks attributed to the PTFE substrate. Additionally, we used Cu K-edge extended X-ray absorption fine structure (EXAFS) to investigate the coordination environment of Cu-MOF and Cu-Carbon-600C. In Figure 1b, the peak around 1.5 Å in the Cu-MOF sample is attributed to Cu–O bonds from the carboxylate group of the *benzene-1,3,5-tricarboxylate* ligand, while the peak around 2.2 Å corresponds to Cu–Cu in the paddlewheel motif.^40^ In contrast, Cu-Carbon-600C shows a small Cu–O peak around 1.5 Å, likely due to oxidation upon air exposure.^41^ However, a prominent Cu–Cu peak around 2.2 Å confirms that the pyrolyzed sample predominantly consists of metallic copper. Next, we performed wavelet analysis on Cu-MOF and Cu-Carbon-600C.

Wavelet analysis was also performed on Cu-MOF and Cu-Carbon-600C. As shown in the wavelet diagrams (Figure 1c,d), Cu-MOF exhibits a wave packet at a distance of around 1.4 Å, with a peak at *k* ≈ 4 Å^–1^, originating from Cu–O bonds, involving lighter atoms. In contrast, Cu-Carbon-600C shows a wave packet at a distance of around 2.4 Å, with a peak at *k* ≈ 7 Å^–1^, contributed by Cu–Cu bonds, involving heavier atoms. From these results, we can deduce that the copper in Cu-MOF is primarily coordinated by Cu–O bonds, while the copper in Cu-Carbon-600C is mostly coordinated by Cu–Cu bonds.

**Figure 2:**
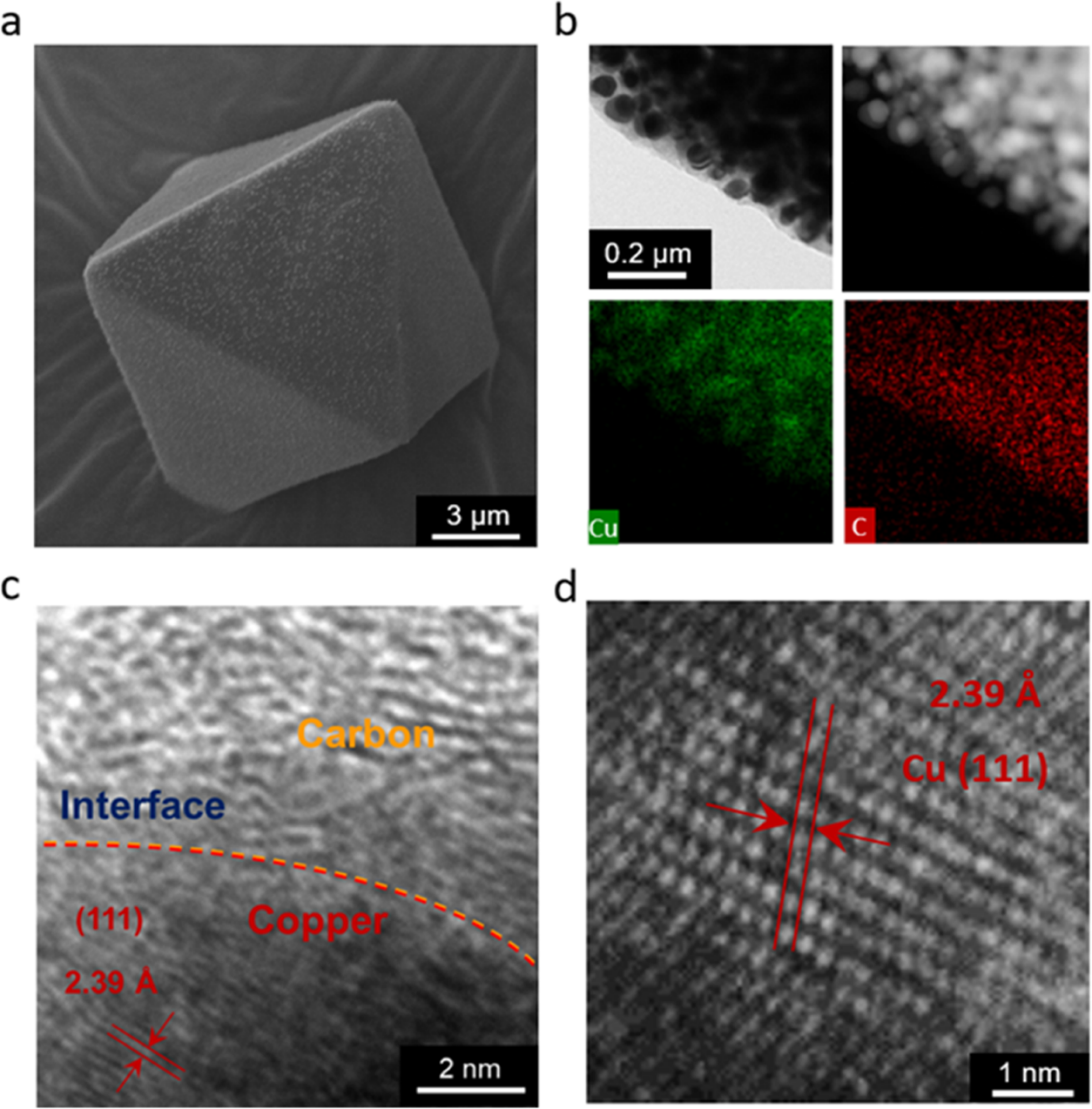
(a)SEM images of Cu-Carbon-600C. (b) TEM images of Cu-Carbon-600C in bright field, dark field and EDS mapping. High resolution transmission electron microscopy image for Cu-Carbon-600C, showing (c) interface between copper and carbon and (d) lattice fringe of copper.

We used scanning electron microscopy (SEM) to identify the morphology of Cu-MOF and Cu-Carbon. As shown in Figure S2, the Cu-MOF sample exhibited an octahedral crystal structure with a particle size of ∼15 μm, consistent with previous reports.^38^ Pyrolyzed samples, treated at temperatures ranging from 400 to 800 °C, displayed a noticeable reduction in particle size, as observed in the SEM images. Despite maintaining their octahedral shape after the pyrolysis, the surface of the samples became distinctly rougher (Figures 2a and S3).

Further analysis using bright-field and dark-field transmission electron microscopy (TEM), along with energy dispersive X-ray spectrometer (EDX), was conducted on the Cu-Carbon-600C sample (Figure 2b). After the pyrolysis process, copper nanoparticles, approximately 60 nm in size, were found to be embedded within the carbon matrix. High-resolution TEM (HRTEM) image of these copper nanoparticles revealed the interface between copper and carbon (Figure 2c) and a well-ordered arrangement corresponding to the (111) facet of metallic copper, with a lattice spacing of 2.39 Å (Figure 2d), confirming the formation of metallic copper following pyrolysis.

Next, we performed Brunauer–Emmett–Teller (BET) analysis on Cu-MOF and Cu-Carbon-600C. As shown in Table S1, Cu-MOF exhibits a high specific surface area of 1234 m^2^/g, attributed to its multicavity structure, which enhances gas adsorption capabilities. In contrast, Cu-Carbon-600C, subjected to thermal pyrolysis, undergoes decomposition of COO^–^ ligands at elevated temperatures, resulting in structural collapse of the MOF framework. This structural alteration significantly reduces the specific surface area to 270 m^2^/g, reflecting the impact of the pyrolysis process on the material’s microenvironment and porosity.

**Figure 3:**
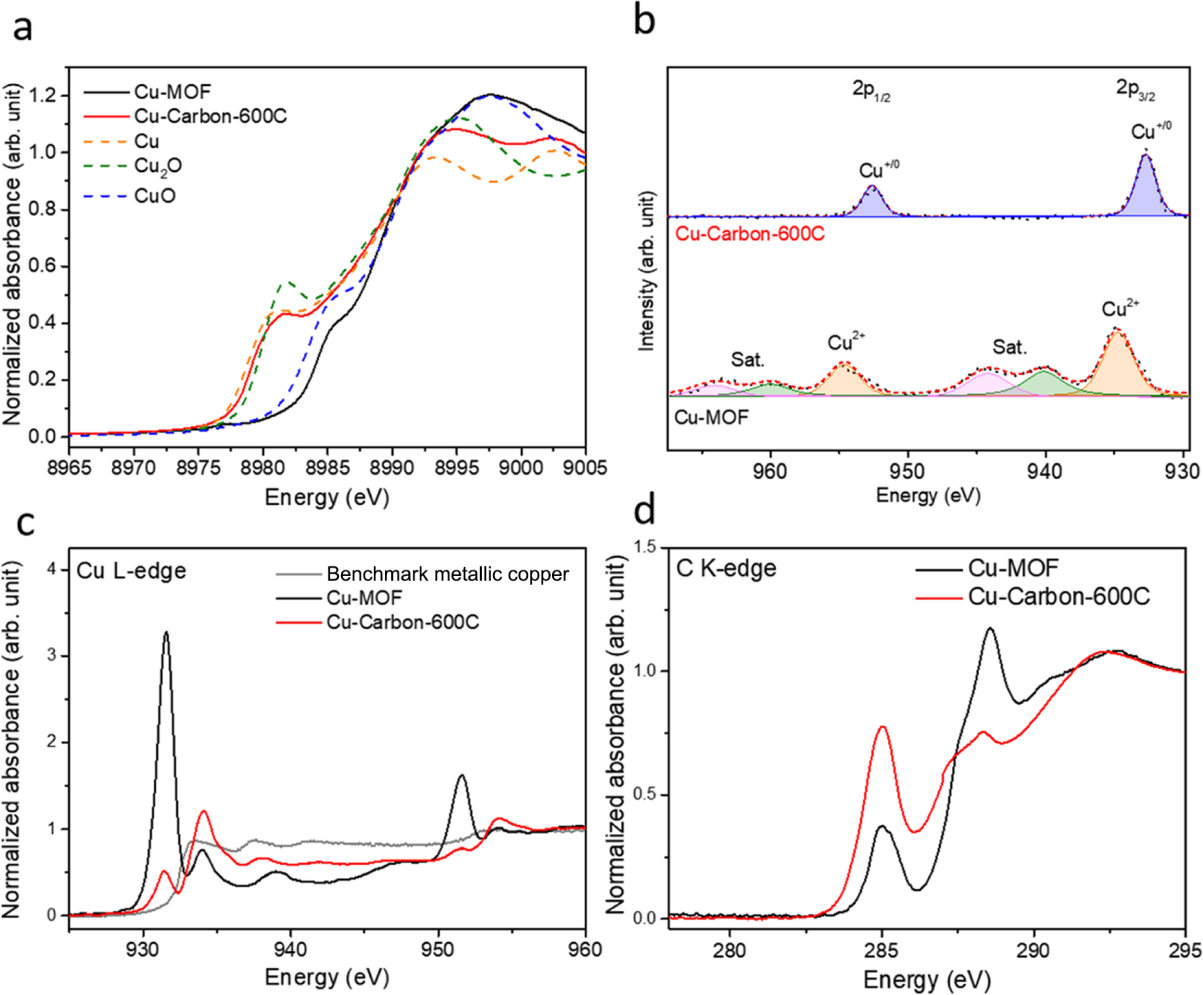
(a) XANES of Cu-MOF, Cu-Carbon-600C, Cu, Cu_2_O and CuO. (b) Cu XPS of Cu-MOF and Cu-Carbon-600C. (c) XAS of Cu L-edge of benchmark metallic copper, Cu-MOF and Cu-Carbon-600C. (d) XAS of C K-edge of Cu-MOF and Cu-Carbon-600C.

We next employed X-ray absorption near-edge structure (XANES) to analyze the valence states of Cu-MOF and Cu-Carbon-600C (Figure 3a). By comparing the results with reference materials such as Cu, CuO, and Cu_2_O, we determined that the cooper in Cu-MOF exhibited a + 2 oxidation state, while after pyrolysis, the copper Cu in Cu-Carbon-600C is reduced to the metallic state. In Figure 3b, the energy range from 930 to 965 eV corresponds to Cu 2p X-ray photoelectron spectroscopy (XPS). For Cu-MOF, the binding energies at 934.7 eV for Cu 2p_3/2_ and 954.6 eV for Cu 2p_1/2_, accompanied by shakeup satellite peaks (940.2, 944.2, 960.1, and 964.1 eV), confirm that the copper is in a + 2 oxidation state.

Conversely, in the Cu-Carbon-600C sample, the binding energies of 932.5 eV for Cu 2p_3/2_ and 954.6 eV for Cu 2p_1/2_ indicate the presence of metallic copper. The absence of satellite peaks in the sample further suggests that the copper crystallites formed during the pyrolysis are of high purity.^42^ In the deconvoluted Cu-Carbon-400C (Figure S4a) we can observe two oxidation states, with peaks at 932.5 and 934.6 eV, corresponding to metallic copper and a minor amount of copper oxides

A comparison of Cu 2p XPS across all pyrolyzed samples (Figure S4a) revealed that as the pyrolysis temperature increased from 400 to 800 °C, the Cu 2p_3/2_ peak shifted from 932.5 to 932.8 eV. This blue shift in binding energy suggests a decrease in electron density at the copper sites with increasing pyrolysis temperature. We also examined the C 1s XPS (Figure S4b), where the 288.8 eV peak in Cu-MOF corresponds to the −COOH group from the *benzene-1,3,5-tricarboxylate* ligand, which disappears after pyrolysis. For the pyrolyzed samples, the C=C binding energy shifts to lower energy as pyrolysis temperature increases, with the peak moving from 285.6 eV in Cu-Carbon-400C shifts to 285.3 eV in Cu-Carbon-800C, indicating increasing electron density in C=C with temperature. This trend, along with the Cu 2p XPS data, indicates that pyrolysis allows for modulation of the electron densities of both copper and C=C sites, thus tuning their electronic structures.

Soft X-ray absorption spectroscopy was also performed to examine the Cu L-edge and C K-edge (Figure 3c,d). Cu-MOF exhibited strong near-edge absorption at 931.5 eV, indicative of a high number of empty d orbitals, corresponding to the higher oxidation state of copper. In contrast, Cu-Carbon-600C showed weaker absorption at 931.5 eV but stronger absorption at 937.5 eV, corresponding to metallic copper with occupied d orbitals.^43^ In comparison, the benchmark metallic copper, with its fully filled d orbitals, lacks a peak at 931.5 eV. Therefore, we speculate that at the Cu–C interface of Cu-Carbon600C, electrons are slightly drawn toward the carbon. In the C K-edge XAS, the peaks at 285 and 288 eV correspond to the transitions of C 1s to C–C π* states and oxygen functional groups in samples, respectively.^44^ As shown in Figure 3d, the pyrolysis of Cu-Carbon-600C led to the removal of oxygen functional groups and an increase in C–C bonding, suggesting that the pyrolysis process carbonized the ligands of Cu-MOF.

**Figure 4:**
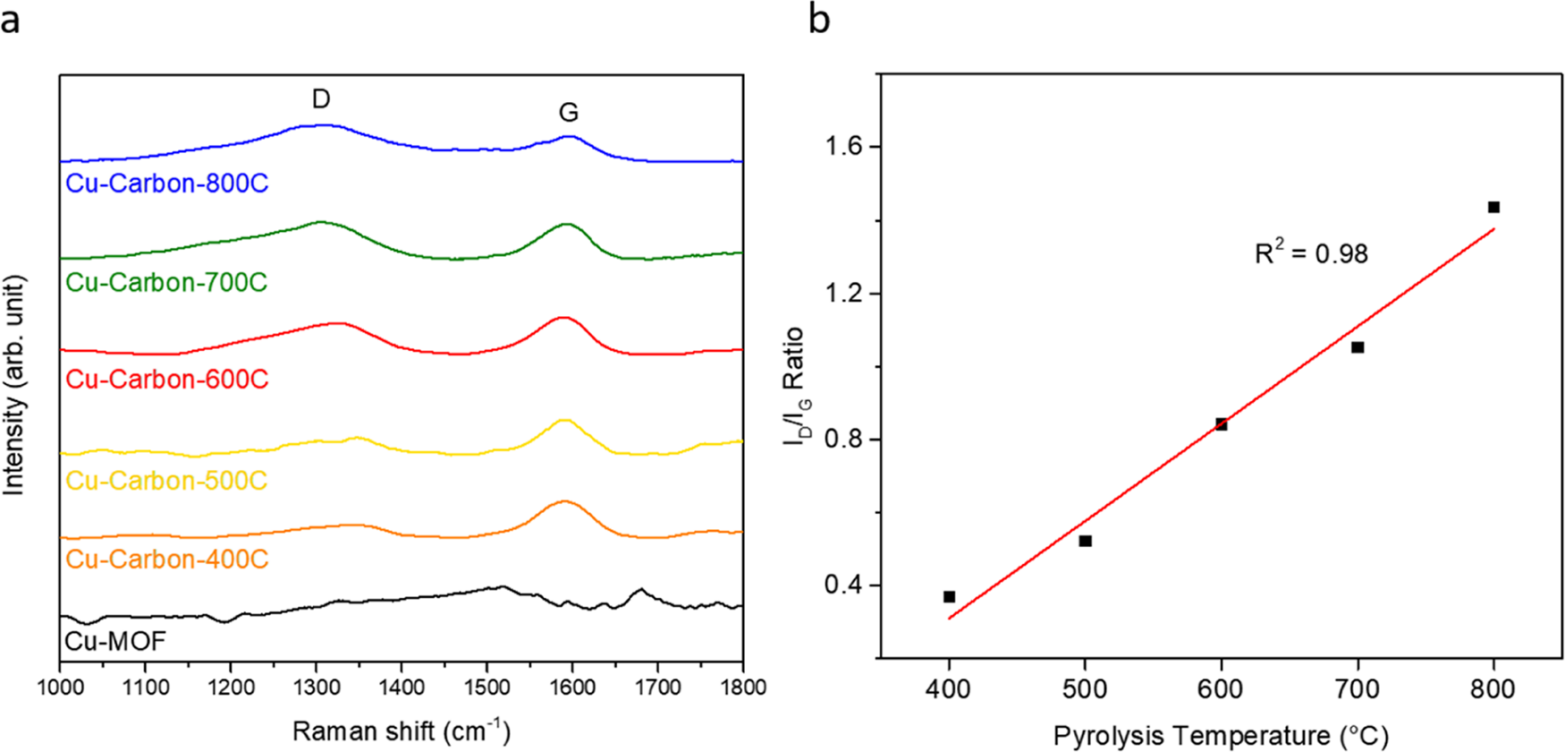
(a) Raman spectra of Cu-MOF and Cu-Carbon at various pyrolysis temperatures. (b) Variation of I_D_/I_G_ ratio with the pyrolysis temperature.

High-resolution TEM images have clearly revealed the interface between the copper nanoparticles and carbon matrix, as well as the lattice spacing of the copper metal nanoparticle in Figure 2c. To further investigate the carbon structure of the samples, we analyzed their Raman spectra, focusing on the D band (∼1350 cm^–1^) and G band (∼1582 cm^–1^). The D band is associated with the vibrations of electron-deficient sp^3^ carbon atoms in defects, while the G band corresponds to the in-plane vibrational mode of the graphitized sp^2^ carbon network.^45,46^ As shown in Figure 4a, the uncarbonized Cu-MOF shows no significant D or G band signals, whereas the pyrolyzed samples display prominent D and G bands. We calculated the intensity ratio of the D and G bands (*I*_D_/*I*_G_) to assess the degree of disorder in the carbon shells surrounding the copper nanoparticles formed during Cu-MOF pyrolysis at various temperatures. An increase in the *I*_D_/*I*_G_ ratio reflects a rise in disorder or defect density.

As shown in Figure 4b, the *I*_D_/*I*_G_ ratio increases from 0.4 to 1.4 as the pyrolysis temperature rises, with an *R*^2^ value of 0.98. This trend aligns with the results reported by Weng et al., who observed a similar increase in disorder in Cu-MOF samples annealed in Ar from 400 to 900 °C, where higher temperatures led to a reduction in graphitic structure.^47^ Similarly, Jalal et al. suggested that a high concentration of copper-based nanoparticles could induce structural distortions in the carbon support, increasing defect density and thereby enhancing D band intensity.^48^ These observation changes in the porous carbon structure during Cu-MOF pyrolysis are consistent with these findings. The *I*_D_/*I*_G_ values influence the conductivity of the carbon materials and therefore the catalytic CO_2_RR performance of the Cu-Carbon catalysts.^49−51^ To characterize the carbon material, we performed measurements in the higher wavenumber range (Figure S5) and observed the absence of the 2D band at 2600 cm^–1^. This finding suggests that pyrolysis at temperature below 800 °C did not result in the formation of graphitic carbon but instead transformed the MOF structure into amorphous carbon. This observation aligns with the finding reported in the previous study.^52^

We next evaluated the electrochemical properties and CO_2_RR activity of the samples. All electrochemical and CO_2_RR tests were conducted in a flow cell, using 1 M KOH aqueous solution as the electrolyte. However, since Cu-MOF is unstable in alkaline conditions (Figure S6), 1.0 M KHCO_3_ was used as the electrolyte in that case. Initially, we assessed the catalyst activity via linear sweep voltammetry (LSV). As shown in Figure S7, the onset potential of the pyrolyzed samples decreased, aligning with the benchmark metallic copper, while the current density increased more steeply at higher applied voltages. These results suggest that the pyrolyzed samples exhibit enhanced CO_2_RR activity compared to Cu-MOF.

Prior to detailed analysis of CO_2_RR activity, we conducted a preliminary screening for the most efficient CO_2_RR catalysts by measuring ethylene production. Figure S8a shows that all pyrolyzed samples achieved higher ethylene Faradaic efficiencies (FE) at every current density compared to Cu-MOF, with Cu-Carbon-600C reaching a maximum ethylene FE of 55.5% at 500 mA cm^–2^, which is a significant improvement over the 36.1% FE of Cu-MOF. Figure S8b highlights the ethylene current density for each sample, where Cu-Carbon-600C achieved an ethylene current density of 277.4 mA cm^–2^ at 500 mA cm^–2^, significantly surpassing the 180.7 mA cm^–2^ exhibited by Cu-MOF.

To provide a more accurate comparison of the catalytic activity of the Cu-Carbon series samples, we performed ICP-MS analysis to determine the weight percentage of copper in each sample, as detailed in Table S2. Additionally, we calculated the C_2_H_4_ turnover frequency (TOF) values for the Cu-Carbon-XC samples at various current densities (Figure S9). The results indicate that Cu-Carbon-600C exhibits the highest TOF among all the catalysts, further corroborating its superior catalytic performance.

Furthermore, double-layer capacitance measurements were performed for the benchmark metallic coper, Cu-MOF, and Cu-Carbon samples to determine the electrochemical active surface areas (ECSA) of the electrodes (Figure S10). As shown in Figure S11a,b the benchmark metallic copper exhibited the largest double-layer capacitance, corresponding to the highest ECSA (36.9 cm^2^). In comparison, Cu-MOF displayed a relatively high ECSA (24.5 cm^2^), whereas the Cu-Carbon samples exhibited the smallest ECSAs, with the values of 10.3, 12.1, 9.7, 11.7, and 12.1 cm^2^ for Cu-Carbon-400C, 500C, 600C, 700C, and 800C, respectively. To enable a more accurate comparison of intrinsic catalytic activity, the LSV curves were normalized using the ECSA values. As shown in Figure S12, the Cu-Carbon-600 sample demonstrated the highest intrinsic activity among the evaluated materials. Based on these results, the optimized Cu-Carbon-600C catalyst was further investigated.

**Figure 5:**
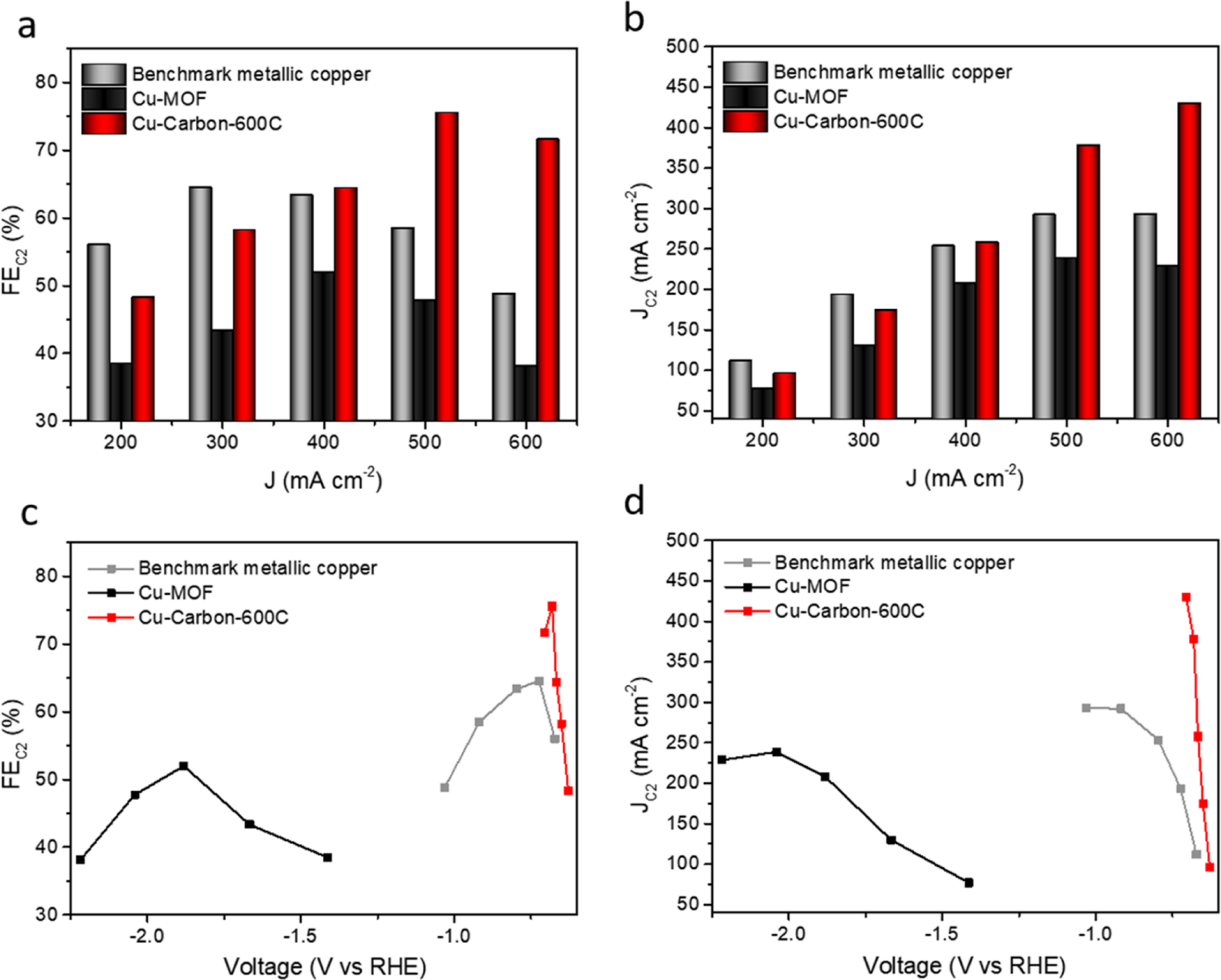
(a) Faradaic efficiency of C_2_ products of benchmark metallic copper, Cu-MOF and Cu-Carbon-600C at various current density. (b) Partial current density of C_2_ products of benchmark metallic copper, Cu-MOF and Cu-Carbon-600C at various current density. (c) Faradaic efficiency of C_2_ products of benchmark metallic copper, Cu-MOF and Cu-Carbon-600C at various applied voltage. (d) Partial current density of C_2_ products of benchmark metallic copper, Cu-MOF and Cu-Carbon-600C at various applied voltage.

In Figure S13, we analyzed the effect of the loading amount of Cu-Carbon-600C on catalytic performance. The loading amounts were varied from 0.2, 0.4, 1.0, 1.4, and 1.8 mg cm^–2^, and the C_2_ Faradaic efficiencies measured at 500 mA cm^–2^ were 65.22, 68.67, 75.58, 70.01, and 69.22%, respectively. From this, it can be concluded that a loading amount of 1.0 mg cm^–2^ is the optimal for CO_2_RR.In Figure 5a, we plot the Faradaic efficiency of C_2_ products (ethylene and ethanol) against current density. Cu-Carbon-600C achieved a C_2_ FE of 75.6% at 500 mA cm^–2^, outperforming both Cu-MOF and benchmark metallic copper, which had C_2_ FEs of 47.8 and 58.5%, respectively. Additionally, we analyzed the partial current density for C_2_ products. As shown in Figure 5b, Cu-Carbon-600C reached a C_2_ partial current density of 353.3 mA cm^–2^ at 500 mA cm^–2^, surpassing both Cu-MOF (238.8 mA cm^–2^) and benchmark metallic copper (292.4 mA cm^–2^). This confirms that Cu-Carbon-600C exhibits superior CO_2_RR activity and C_2_ selectivity compared to Cu-MOF and benchmark metallic copper. Faradaic efficiencies for different products across various current densities are provided in Table S3. Additionally, to confirm that the products are derived exclusively from CO_2_, we performed isotopic analysis by conducting electrocatalytic reactions using C^13^-labeled CO_2_ and collected the liquid products for mass spectrometry analysis. As shown in Figure S14, the results reveal characteristic fragments of ^13^C ethanol (*m*/*z* = 32, 47), in contrast to the characteristic fragments of ^12^C ethanol (*m*/*z* = 31, 45). This unequivocally confirms that the CO_2_RR products originate from CO_2_.

We then examined the voltage dependence of CO_2_RR catalytic performance. As shown in Figure 5c,d, Cu-Carbon-600C required an applied voltage of only −0.68 V vs RHE to achieve a C_2_ partial current density of 353.3 mA cm^–2^ with a C2 FE of 70.7%. Furthermore, under an identical applied voltage of −0.67 V vs RHE, Cu-Carbon-600C demonstrated a C_2_ partial current density of 257.1 mA cm^–2^ with a C_2_ FE of 64.5%, significantly outperforming benchmark metallic copper, which exhibited a C_2_ partial current density of 112.1 mA cm^–2^ with a C_2_ FE of 56.0%. In contrast, both Cu-MOF and benchmark metallic copper required higher applied voltages to achieve their respective optimal C_2_ selectivity, with lower C_2_ partial current densities compared to Cu-Carbon-600C. These results underscore that Cu-Carbon-600C is a more efficient and energy-saving catalyst with enhanced selectivity, surpassing prior reports (Table S4).^53−58^ We also conducted a long-term stability test (Figure S15) to evaluate the durability of Cu-Carbon-600C under continuous operation. The results demonstrate that Cu-Carbon-600C remains stable at a current density of 500 mA cm^–2^ for at least 10 h, with the C_2_ Faradaic efficiency consistently exceeding 70%.

We then focused on using electrochemical impedance spectroscopy (EIS) to assess the charge transfer capabilities involved in electrocatalytic CO_2_RR. Figure S16 displays the Nyquist plots for benchmark metallic copper, Cu-MOF and Cu-Crabon-400C, Cu-Crabon-500C, Cu-Crabon-600C, Cu-Crabon-700C and Cu-Crabon-800C, respectively. These plots show that as the applied voltage increases, the semicircles gradually shrink, indicating the activation of the catalyst. The semicircles at an applied voltage of −0.8 V (vs RHE) were fitted using an equivalent circuit model, and the results (Figures 7a,b and S17) demonstrated good fitting quality. Cu-Carbon-600C exhibited lower series resistance (*R*_S_ = 8.474 Ω) and charge transfer resistance (*R*_CT_ = 5.004 Ω) compared to benchmark metallic copper (*R*_S_ = 10.64 Ω and *R*_CT_ = 5.976 Ω) and Cu-MOF (*R*_S_ = 22.67 Ω and *R*_CT_ = 21.76 Ω). It is worth noting that all the samples after pyrolysis show a significant decrease in *R*_S_ and *R*_CT_ compared to Cu-MOF, indicating lower overall resistance and faster electron transfer, further supporting its superior CO_2_RR activity.

**Figure 6:**
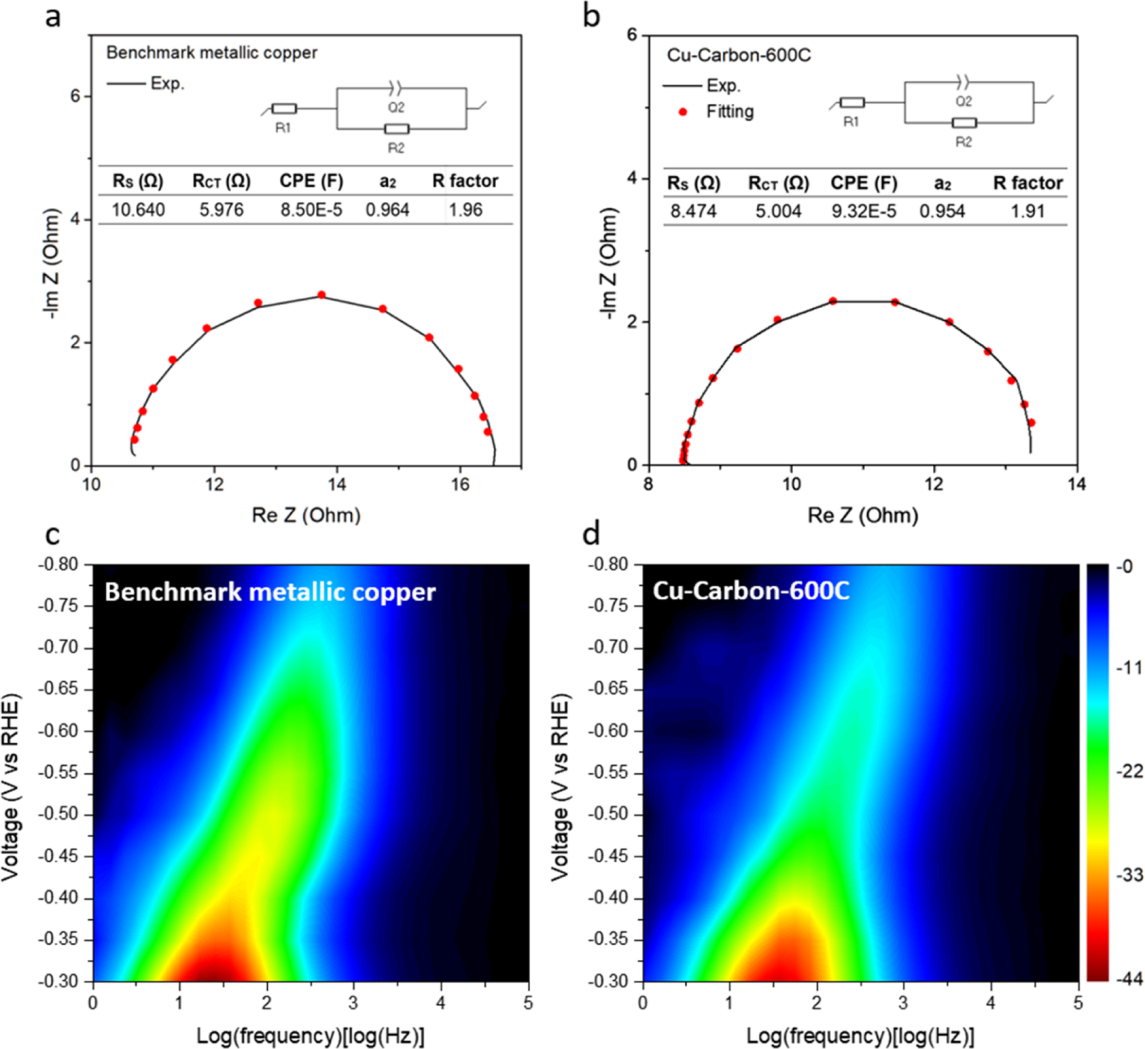
(a) Equivalent circuit for fitting the Nyquist plot and Nyquist plot of benchmark metallic copper. (b) Equivalent circuit for fitting the Nyquist plot and Nyquist plot of Cu-Carbon- 600C. (c) 2D Bode plot of benchmark metallic copper. (d) 2D Bode plot of Cu-Carbon-600C.

Next, we analyzed the Bode plots (Figure S18) by plotting the phase angle against the logarithm of frequency. We observed that as the applied voltage increased, the phase angle decreased, suggesting a reduction in charge transfer resistance and an enhancement in catalyst activity. The 2D Bode plots (Figures 6c,d and S19) further illustrate that the phase angle of Cu-Carbon-600C exhibits greater responsiveness to voltage changes compared to benchmark metallic copper and Cu-MOF, demonstrating superior charge transfer capabilities, which positively impacts CO_2_RR. EIS analysis revealed that Cu-Carbon-600C exhibits lower resistance and enhanced charge transfer capabilities compared to benchmark metallic copper and Cu-MOF. This improvement is likely attributed to the carbon matrix formed during pyrolysis, which promotes efficient charge transport between the copper nanoparticles and the surrounding carbon material, thereby enhancing catalytic performance.

We conducted postreaction analyses to determine whether the structure and morphology of the catalyst changed after the reaction. First, post-XRD patterns were collected, as shown in Figure S20. A peak at 43.4° was observed, corresponding to the (111) facet of metallic copper, indicating that the copper in Cu–Carbon–600C remained in its metallic state even after high-current electrochemical catalysis.

Next, post-SEM analysis (Figure S21) revealed that while the surface of Cu–Carbon–600C became rougher after electrochemical catalysis, it retained its complete octahedral structure, similar to its morphology prior to the reaction. To gain further insights, we performed postreaction TEM analysis combined with EDX spectroscopy (Figure. S22). The results showed that Cu–Carbon–600C maintained its structure, with copper particles embedded within the carbon matrix after the reaction. However, some copper nanoparticles exhibited signs of aggregation, resulting in increased particle sizes. EDX mapping provided clear evidence of copper nanoparticles encapsulated by carbon. From these observations, we conclude that the morphology and structure of Cu–Carbon–600C did not undergo significant changes after the reaction. Furthermore, the carbon-encapsulated copper microenvironment created during the pyrolysis process remained intact throughout the catalytic process.

**Figure 7:**
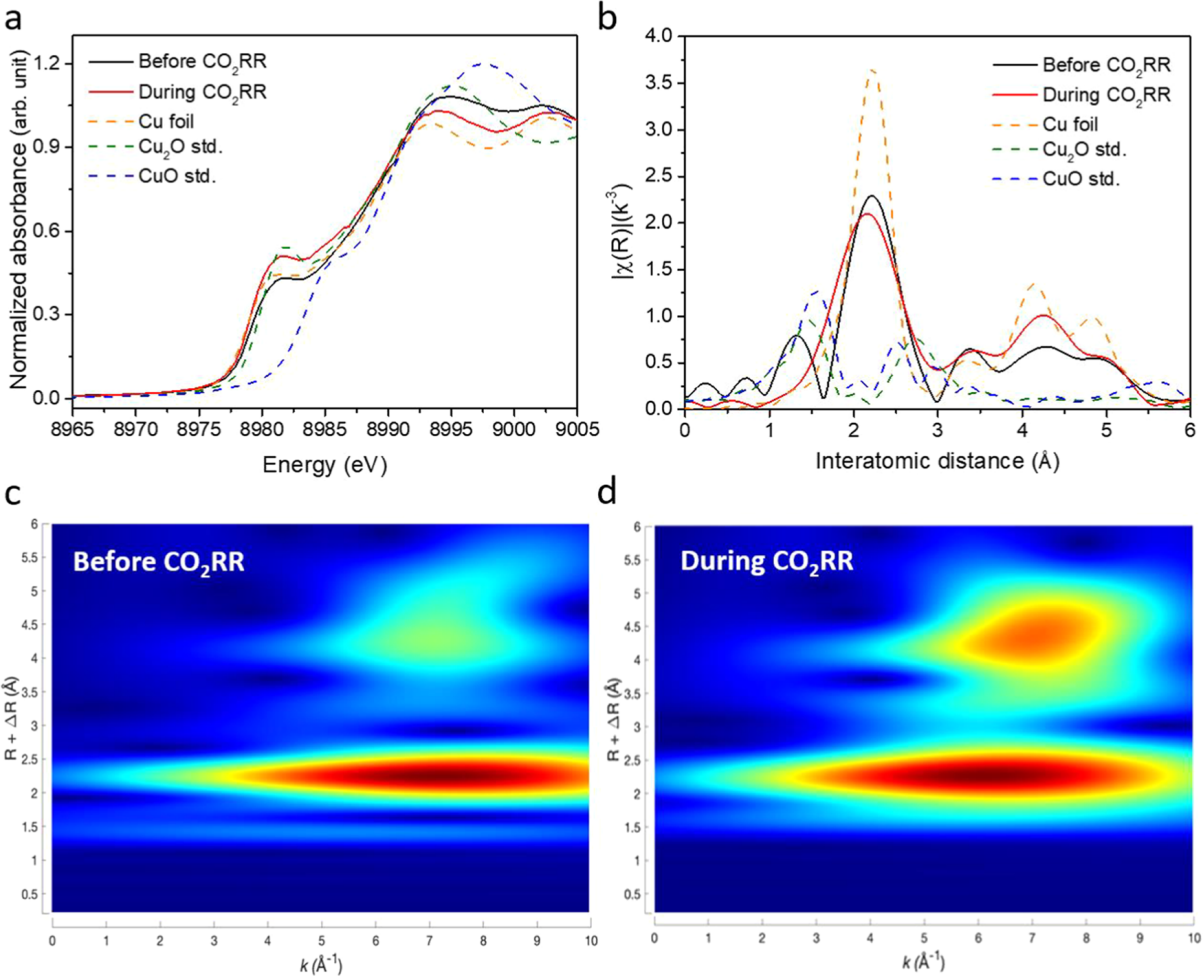
(a) In-situ XANES of Cu-Carbon-600C before and during CO_2_RR. (b) In-situ EXAFS of Cu-Carbon-600C before and during CO_2_RR. Wavelet analysis of Cu-Graphene-600C (c) before CO_2_RR and (d) after CO_2_RR.

We then performed in situ XAS experiments using a specially designed flow cell to observe the states and structural changes of the catalyst during the CO_2_RR process.^59,60^ As shown in Figure 7a (in situ XANES), the catalyst exhibits a metallic copper state both before and during CO_2_RR. Figure S23 was generated by fitting the valence state against the absorption energy. From the plot, it is evident that the binding of copper with ligands in Cu-MOF yields a Cu oxidation state of +1.99, closely resembling that of CuO. For Cu-Carbon-600C, the oxidation state prior to CO_2_RR is +0.01, indicating a state very similar to metallic copper. During the CO_2_RR reaction, the copper in Cu-Carbon-600C is fully reduced to a zerovalent metallic state.

In Figure 7b (in situ EXAFS), prior to CO_2_RR, a peak at 1.4 Å corresponds to Cu–O formation, likely resulting from partial oxidation during the preparation of the gas diffusion electrode via spray coating. However, during CO_2_RR, the Cu–O peak disappears, indicating its reduction to metallic Cu–Cu, with the peak shifting to 2.2 Å. Furthermore, we analyzed the changes in the coordination number (C.N.) of Cu-MOF and Cu-Carbon-600C before and during the catalytic reaction through EXAFS fitting. The fitting results and associated quality metrics are presented in Figure S24 and Table S5. Following pyrolysis, Cu-MOF transitions from a Cu–O coordination environment to a Cu–Cu coordination environment in Cu-Carbon-600C. During the electrochemical catalytic reaction, the Cu–Cu coordination number remained nearly unchanged, suggesting that the catalyst structure remains stable throughout the reaction process.

To further validate these bonding changes, we conducted in situ XAS wavelet analysis. Although a weak Cu–O wave packet appears around 1.4 Å in Figure 7c, Figure 7d clearly shows the presence of metallic copper structures during CO_2_RR, with the Cu–O bond absent. This confirms that the copper involved in the reaction remains in its metallic form throughout the CO_2_RR process. Consequently, the microenvironment surrounding copper plays a pivotal role in determining CO_2_RR performance, including selectivity, product current density, and the required voltage.

**Figure 8:**
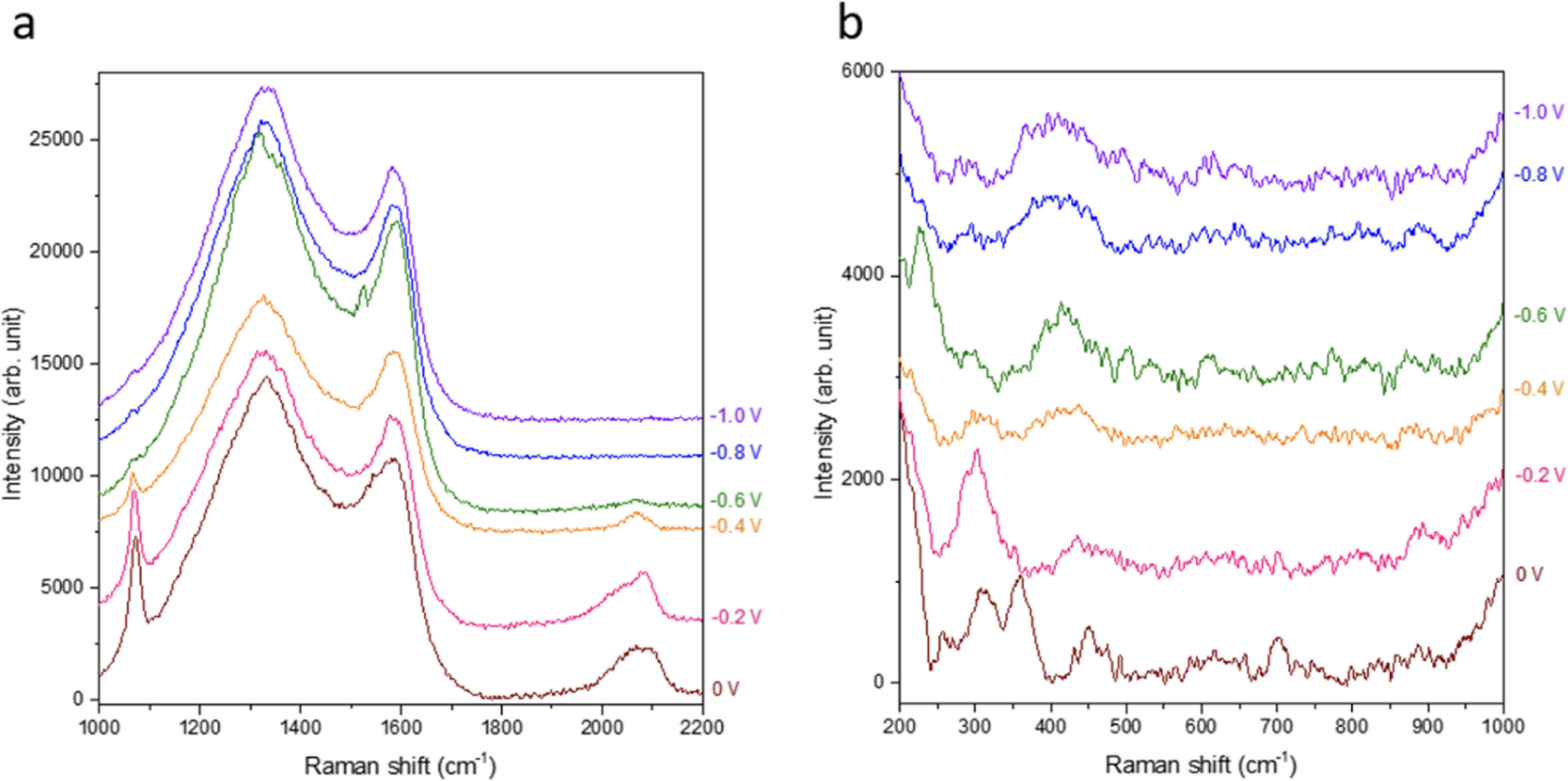
In-situ Raman spectra for Cu-Carbon-600C recorded at various potentials during CO_2_RR: (a) spectral region from 200 to 1000 cm^–1^ and (b) spectral region from 1000 to 2200 cm^–1^.

In-situ Raman spectroscopy was employed to investigate crucial intermediates involving in the CO_2_RR process, with the results presented in Figure 8. In Figure 8a. a peak at 364 cm^–1^,^61^ corresponding to the restricted rotation of Cu–CO stretching, was observed at 0 V on Cu-Carbon-600C. A higher intensity of this Cu–CO stretching band indicates a greater coverage of CO intermediates, which facilitates C–C coupling.^62^ Additionally, a peak at approximately 300 cm^–1^, corresponding to the Cu–CO frustrated rotation,^61,63−65^ was detected at 0 V on Cu-Carbon-600C. However, the intensity of Cu-CO-related peaks on Cu-Carbon-600C was lower, likely due to a reduced Cu-CO concentration associated with a smaller active surface area.

Figure 8b highlights a Raman peaks at 1068 cm^–1^, attributed to the vibration of CO_3_^2–^ which was detected at 0 V on Cu-Carbon-600C.^66^ A peak at approximately 2068 cm^–1^, corresponding to C≡O stretching on Cu, was also observed. This peak can be deconvolved into top-bound CO and bridge-bound CO, suggesting that the pathway for generating C_2+_ products was active.^67^ Additionally, a distinct peak at 1545 cm^–1^ appearing at −0.6 V on Cu-Carbon-600C, was attributed to a critical intermediates for C_2+_ products.^67^ This finding indicates that the copper–carbon interface in Cu-Carbon-600C creates a favorable microenvironment for stabilizing intermediates, thereby enhancing the production of C_2+_ products.

## 4 Conclusions

In this study, we developed a highly efficient Cu-Carbon catalyst for carbon dioxide reduction reaction to C_2_ products. It achieved a Faradaic efficiency of 70.7% for C_2_ products and a C_2_ current density of 353.5 mA cm^–2^ at an applied voltage of −0.68 V vs RHE, outperforming both benchmark metallic copper and Cu-MOF. Raman spectroscopy and XPS analyses revealed structural and electronic interactions between the carbon and copper under various pyrolysis temperature, while electrochemical impedance spectroscopy demonstrated that the copper–carbon composite significantly improved overall conductivity and charge transport capabilities. X-ray absorption spectroscopy confirmed that the copper in the composite remained in a metallic state, with in situ X-ray absorption spectroscopy further validating the active copper maintaining its metallic state during CO_2_RR. These findings offer valuable insights for advancing industrial carbon dioxide reduction, promoting lower carbon emissions, and supporting sustainable development.

## Data Availability

The data supporting this study are available in the paper and the Supporting Information. All other relevant source data are available from the corresponding authors upon reasonable request.

## Supporting Information Available

Additional XRD patterns, SEM images, XPS spectra, Raman spectra, digital pictures, Linear sweep voltammetry, Faradaic efficiency, Turnover frequency, cyclic voltammetry, GCMS spectroscopy, Stability test, Nyquist plots, Bode plots, postreaction XRD, postreaction SEM, postreaction TEM, Valence state fitting, EXAFS fitting, and tables of BET surface area, Copper weight percentage, Faradaic efficiencies of CO_2_RR products, and EXAFS fitting (PDF)
