# Supplementary material for: Microenvironment Matters: Copper–Carbon Composites Enable a Highly Efficient Carbon Dioxide Reduction Reaction to C2 Products
Source: ACS Appl Mater Interfaces. 2025 Feb 4;17(6):9378–90. doi: 10.1021/acsami.4c20586 (PMC11826886; doi:10.1021/acsami.4c20586)
Supplement: Supplementary file 1 — am4c20586_si_001.pdf [file am4c20586_si_001.pdf]

Supporting Information

Microenvironment Matters: Copper-Carbon Composites Enable Highly Efficient Carbon Dioxide Reduction Reaction to C_2 Products

Yu-Jhih Shen,[1] Yung-Hsi Hsu,[1] Yu-Chia Chang,[1] Jian-Jie Ma,[1] Kang-Shun Peng,[1] Ying-Rui Lu,[3] Shao-Hui Hsu,[4] Sung-Fu Hung[1,2*]

[1]*Department of Applied Chemistry and Center for Emergent Functional Matter Science, National Yang Ming Chiao Tung University, Hsinchu 300, Taiwan.*

[2]*Department of Medicinal and Applied Chemistry, Kaohsiung Medical University, Kaohsiung 807, Taiwan.*

[3]*National Synchrotron Radiation Research Center, Hsinchu 300, Taiwan*

[4]*Taiwan Semiconductor Research Institute, National Applied Research Laboratories, Hsinchu 300, Taiwan.*

Email: sungfuhung@nycu.edu.tw

This file includes:

Supplementary Figures 1 to 24.

Supplementary Tables 1 to 5.

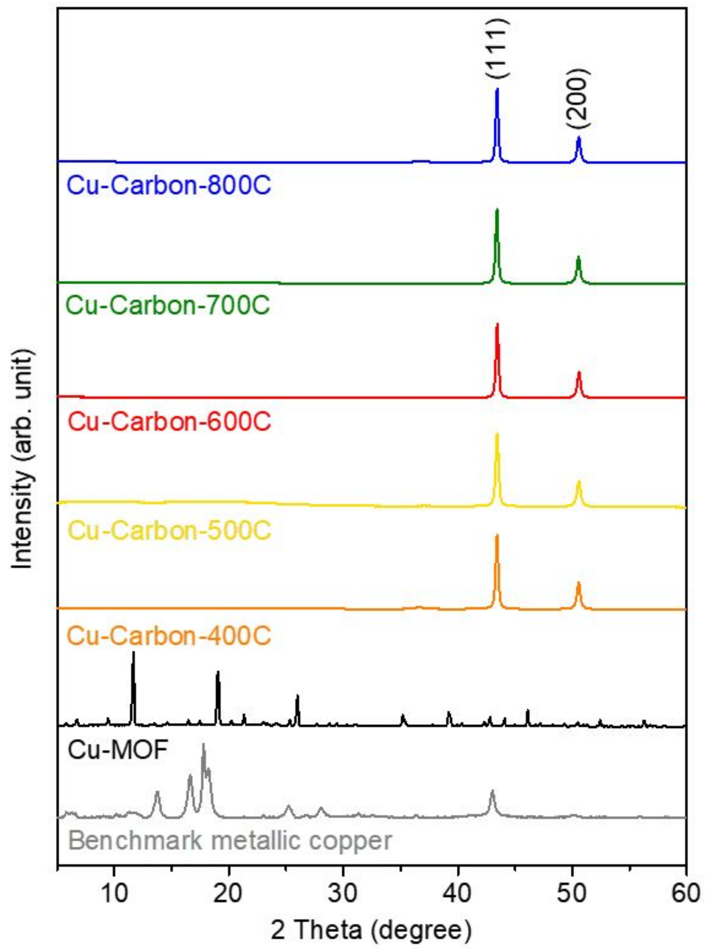

Figure S1. X-ray diffraction patterns of Sputter Cu, Cu-MOF, Cu-Carbon-400C, Cu-Carbon-500C, Cu-Carbon-600C, Cu-Carbon-700C, and Cu-Carbon-800C.

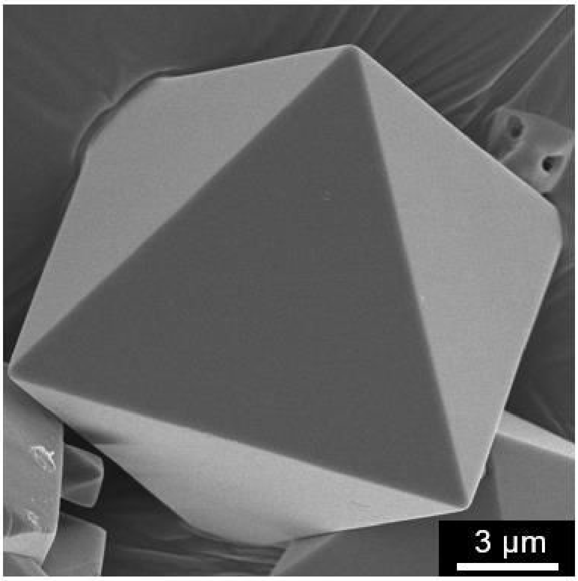

Figure S2. SEM images of Cu-MOF

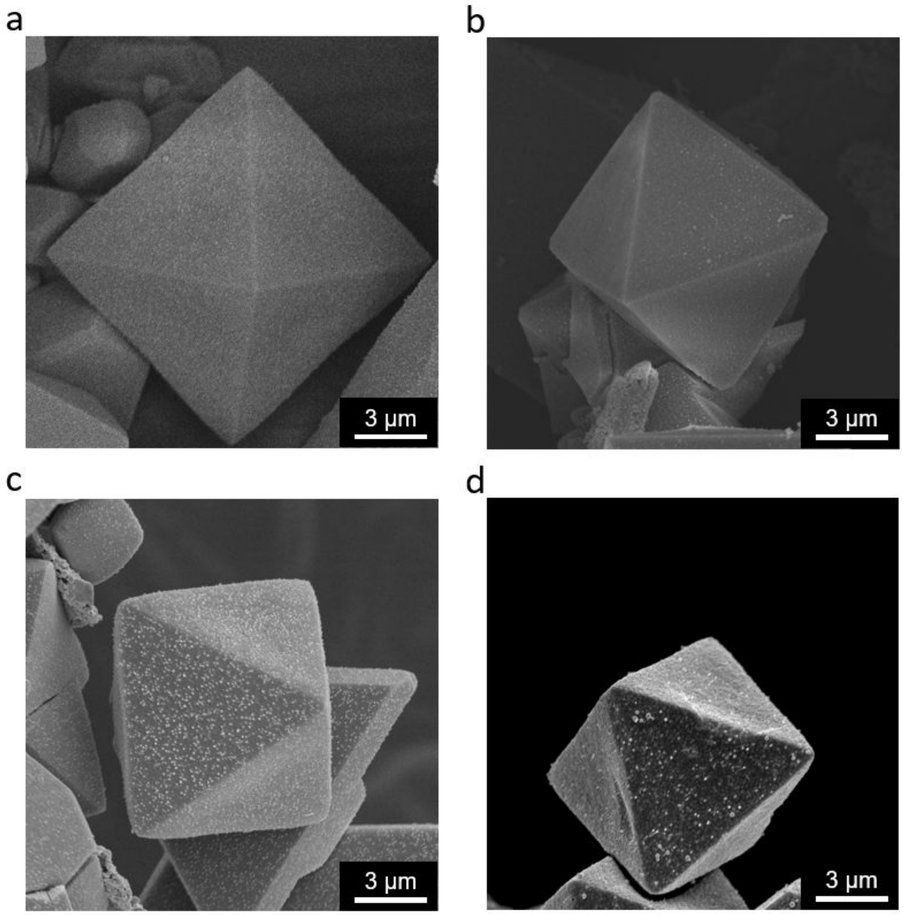

Figure S3. Scanning electron microscopy images of (a) Cu-Carbon-400C, (b) Cu-Carbon-500C, (c) Cu-Carbon-700C and (d) Cu-Carbon-800C.

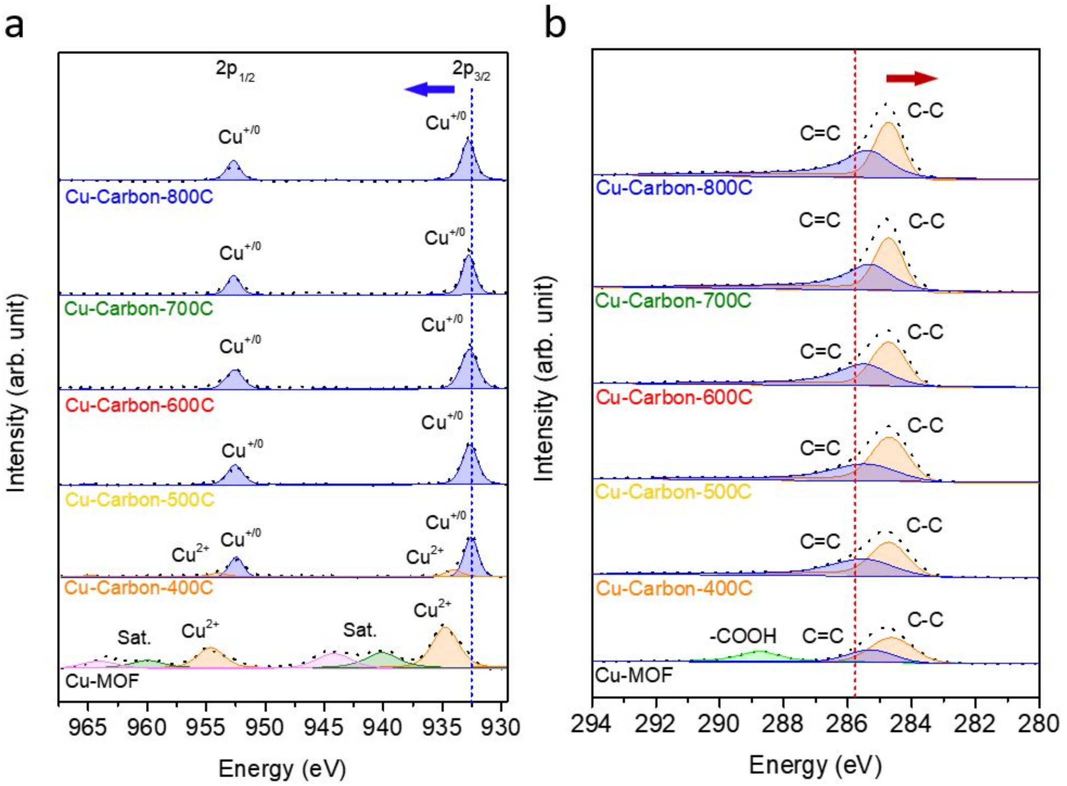

Figure S4. (a) X-ray photoelectron spectroscopy of Cu $2p_{3/2}$ and $2p_{1/2}$ for Cu-MOF, Cu-Carbon-400C, Cu-Carbon-500C, Cu-Carbon-600C, Cu-Carbon-700C, and Cu-Carbon-800C. (b) X-ray photoelectron spectroscopy of C 1S of Cu for Cu-MOF, Cu-Carbon-400C, Cu-Carbon-500C, Cu-Carbon-600C, Cu-Carbon-700C, and Cu-Carbon-800C.

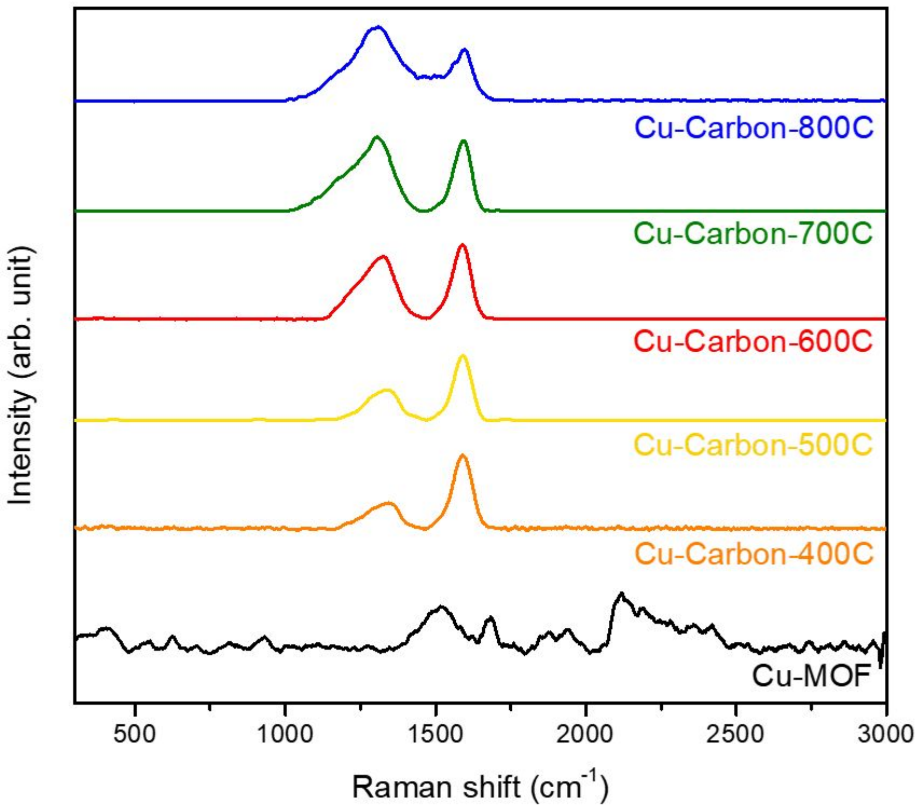

Figure S5. Raman spectra of Cu-MOF and Cu-Carbon at various pyrolysis temperatures.

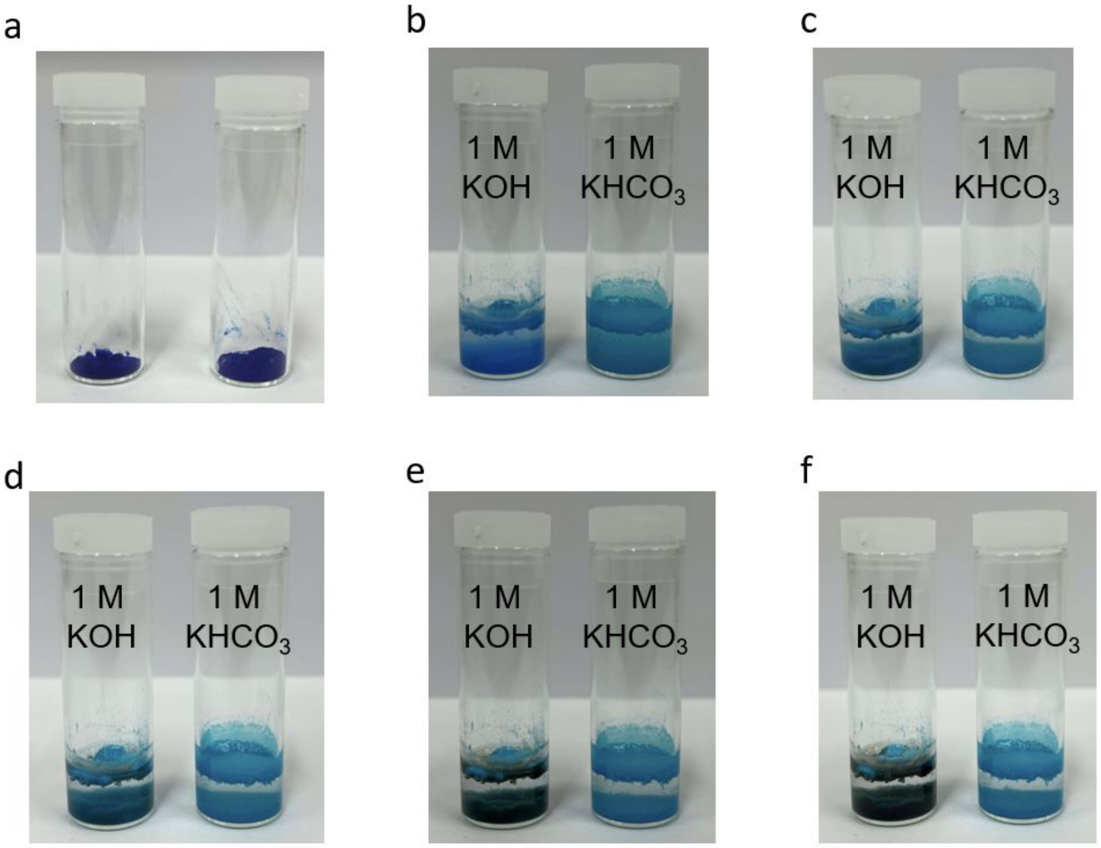

Figure S6. Pictures of Cu-MOF in (a) air and in 1M KOH, 1 M $HKCO_3$ for (b) 0 min, (c) 1 min, (d) 3 min, (e) 5 min, and (f) 15 min.

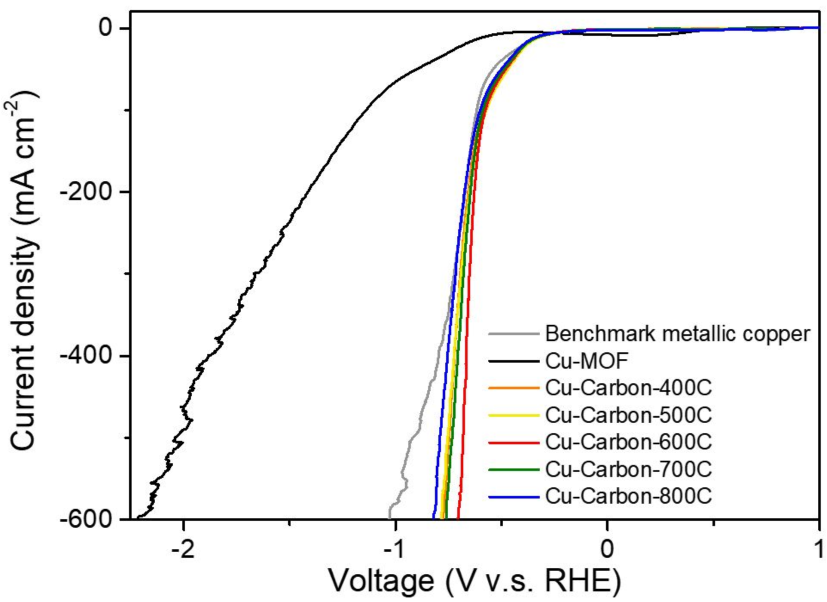

Figure S7. Linear sweep voltammetry of Benchmark metallic copper, Cu-MOF, Cu-Carbon-400C, Cu-Carbon-500C, Cu-Carbon-600C, Cu-Carbon-700C, and Cu-Carbon-800C.

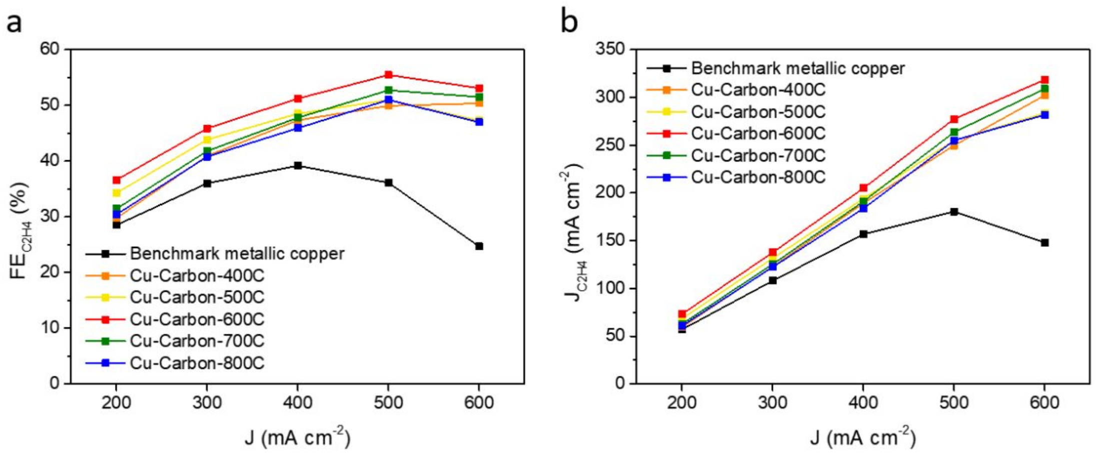

Figure S8. (a) Faradaic efficiency of ethylene of Cu-MOF, Cu-Carbon-400C, Cu-Carbon-500C, Cu-Carbon-600C, Cu-Carbon-700C, and Cu-Carbon-800C at various current densities. (b) Ethylene current density of Cu-MOF, Cu-Carbon-400C, Cu-Carbon-500C, Cu-Carbon-600C, Cu-Carbon-700C, and Cu-Carbon-800C at various current densities.

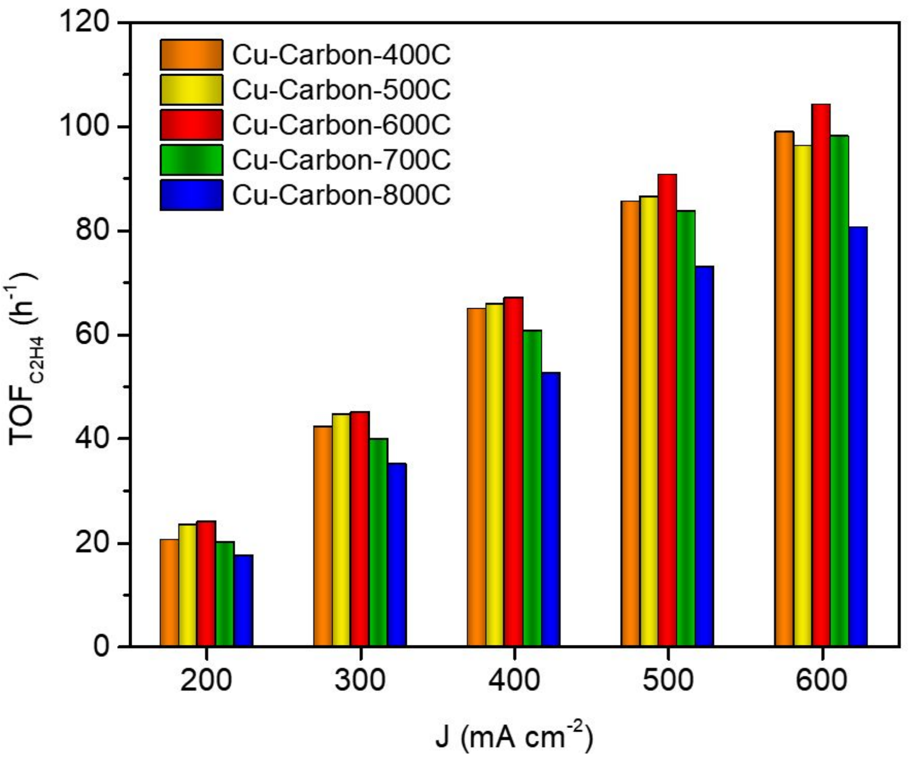

Figure S9. Turnover frequency of ethylene of Cu-Carbon-400C, Cu-Carbon-500C, Cu-Carbon-600C, Cu-Carbon-700C and Cu-Carbon-800C versus current density.

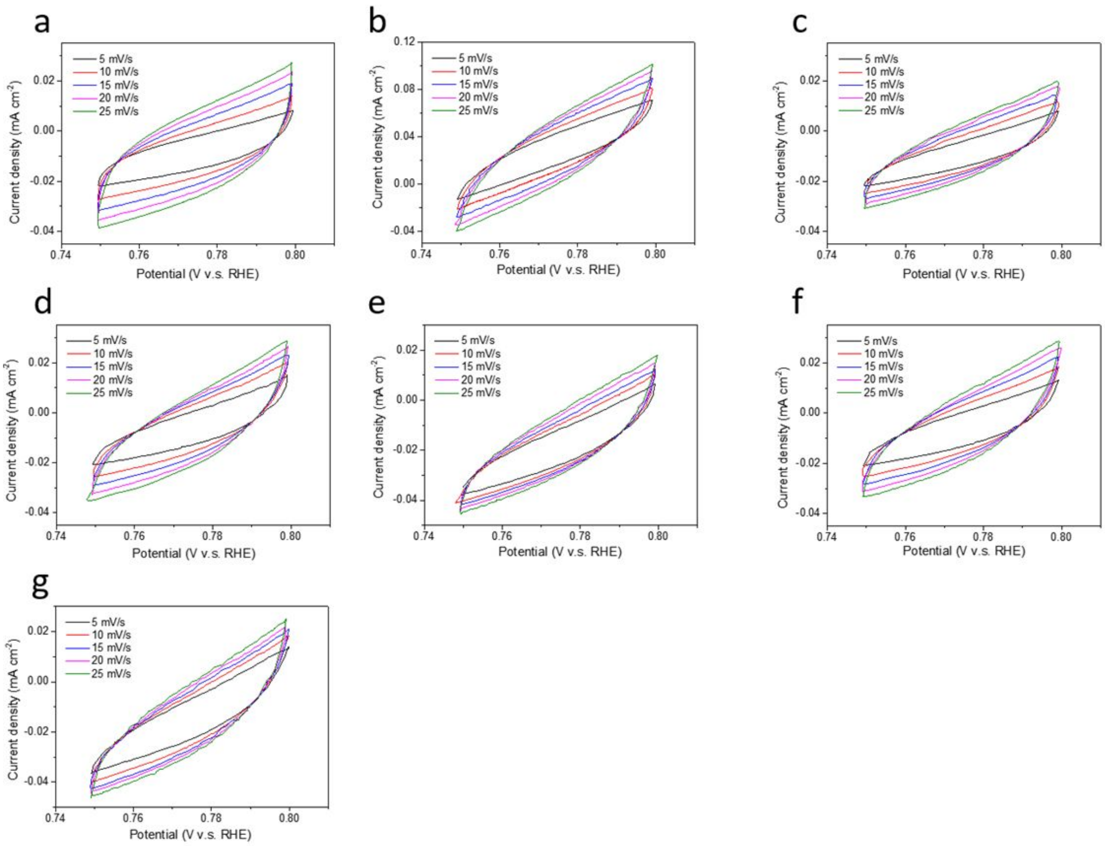

Figure S10. CVs taken over a range of scan rates of (a) benchmark metallic copper, (b) Cu-MOF, (c) Cu-Carbon-400C, (d) Cu-Carbon-500C, (e) Cu-Carbon-600C, (f) Cu-Carbon-700C and (g) Cu-Carbon-800C.

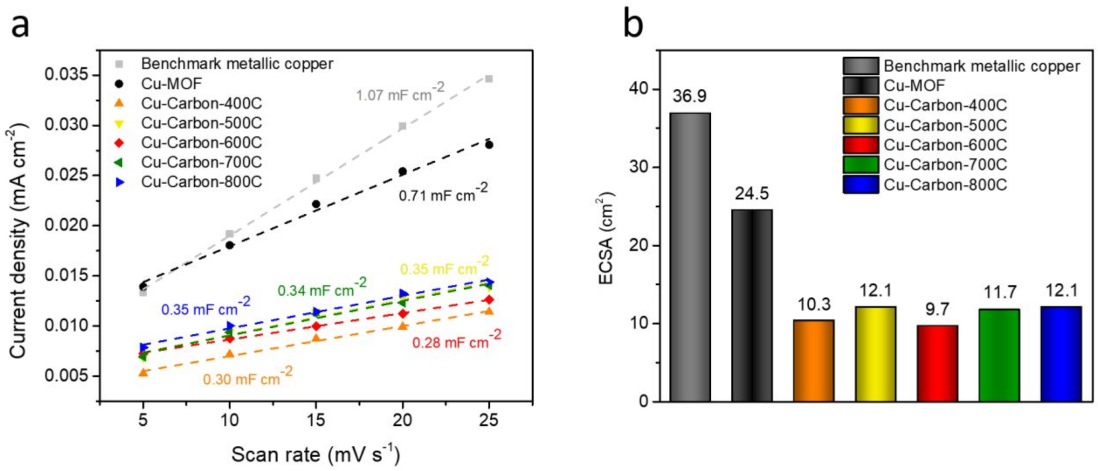

Figure S11. (a) Measured double layer charging current densities vs. scan rates for benchmark metallic copper, Cu-MOF, Cu-Carbon-400C, Cu-Carbon-500C, Cu-Carbon-600C, Cu-Carbon-700C and Cu-Carbon-800C. (b) The specific surface areas of benchmark metallic copper, Cu-MOF, Cu-Carbon-400C, Cu-Carbon-500C, Cu-Carbon-600C, Cu-Carbon-700C and Cu-Carbon-800C.

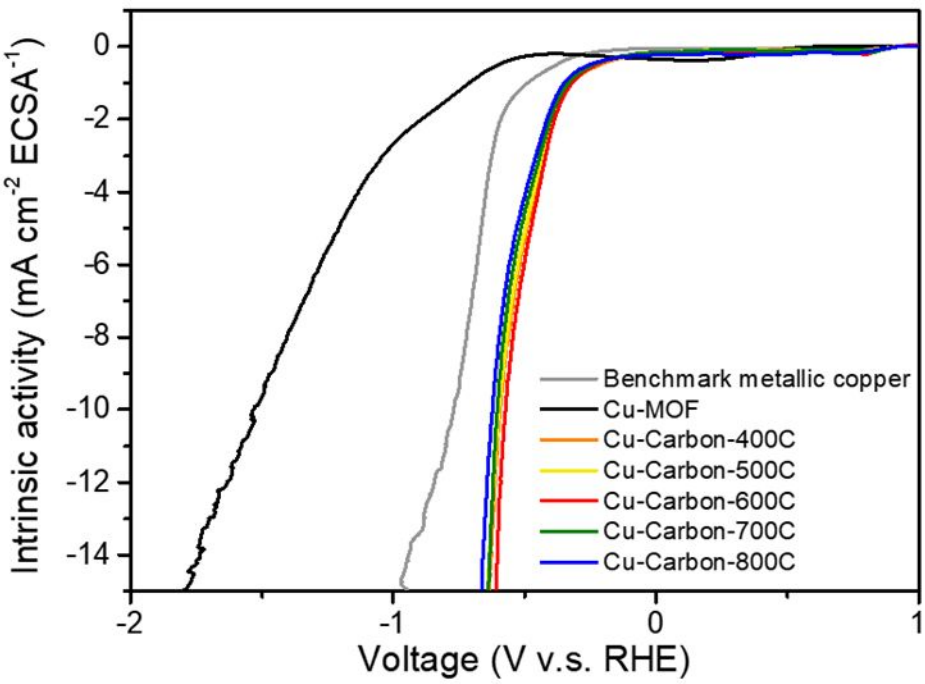

Figure S12. Intrinsic activity of benchmark metallic copper, Cu-MOF, Cu-Carbon-400C, Cu-Carbon-500C, Cu-Carbon-600C, Cu-Carbon-700C and Cu-Carbon-800C.

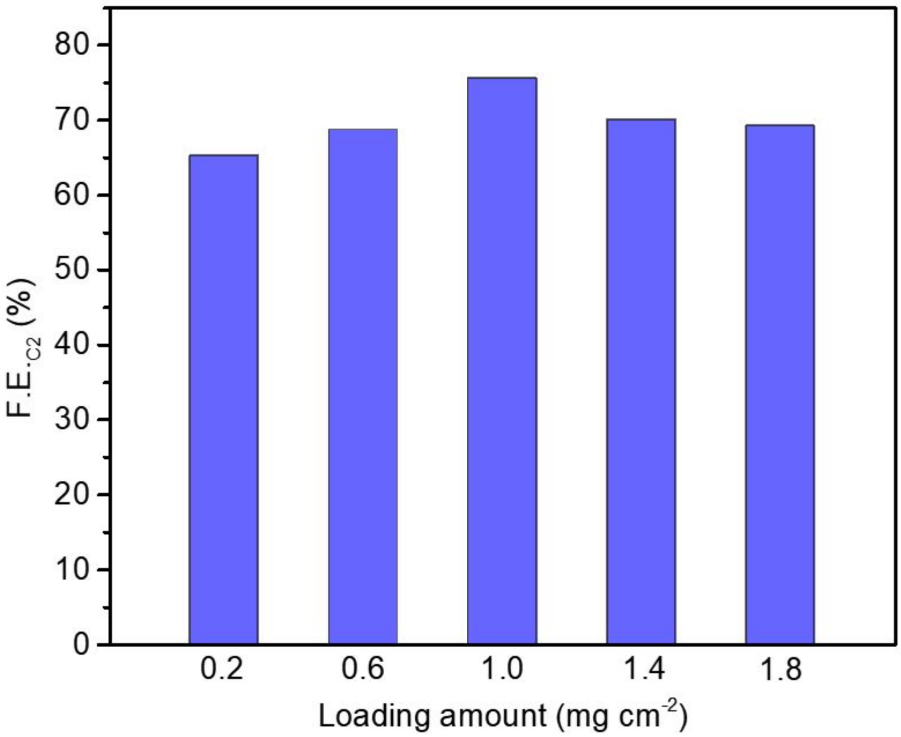

Figure S13. C_2 Faradaic efficiency of Cu-Carbon-600C varying different loading amount.

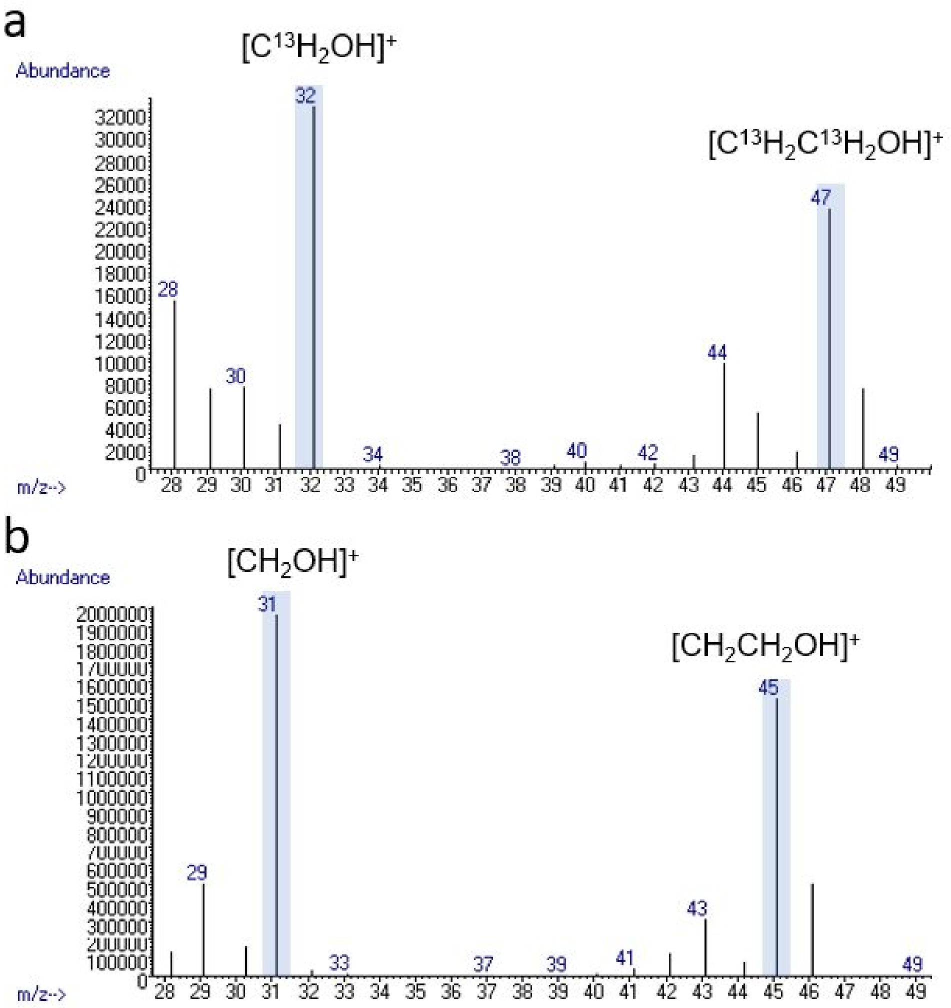

Figure S14. GC-MS analyses of C_2H_5OH produced from CO_2RR on Cu–Carbon–600C using (a) $^{13}CO_2$ and (b) $^{12}CO_2$ as feedstock.

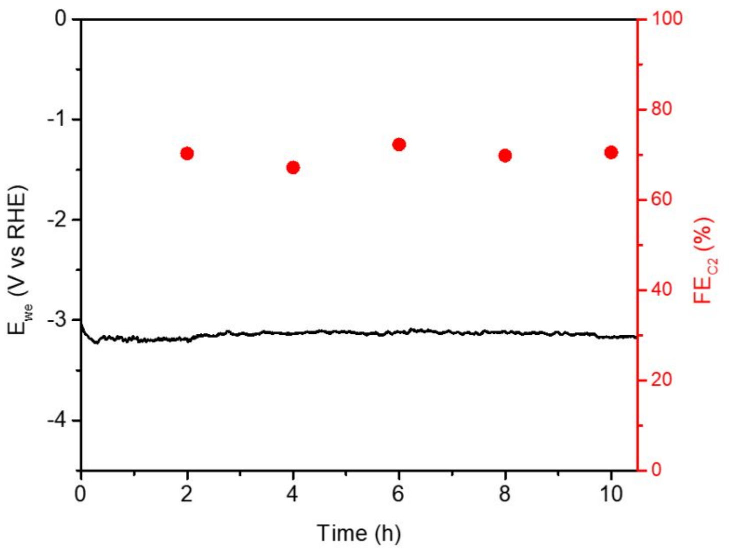

Figure S15. Stability test of Cu-Carbon-600C under 500 mA cm^{-2} in flow cell.

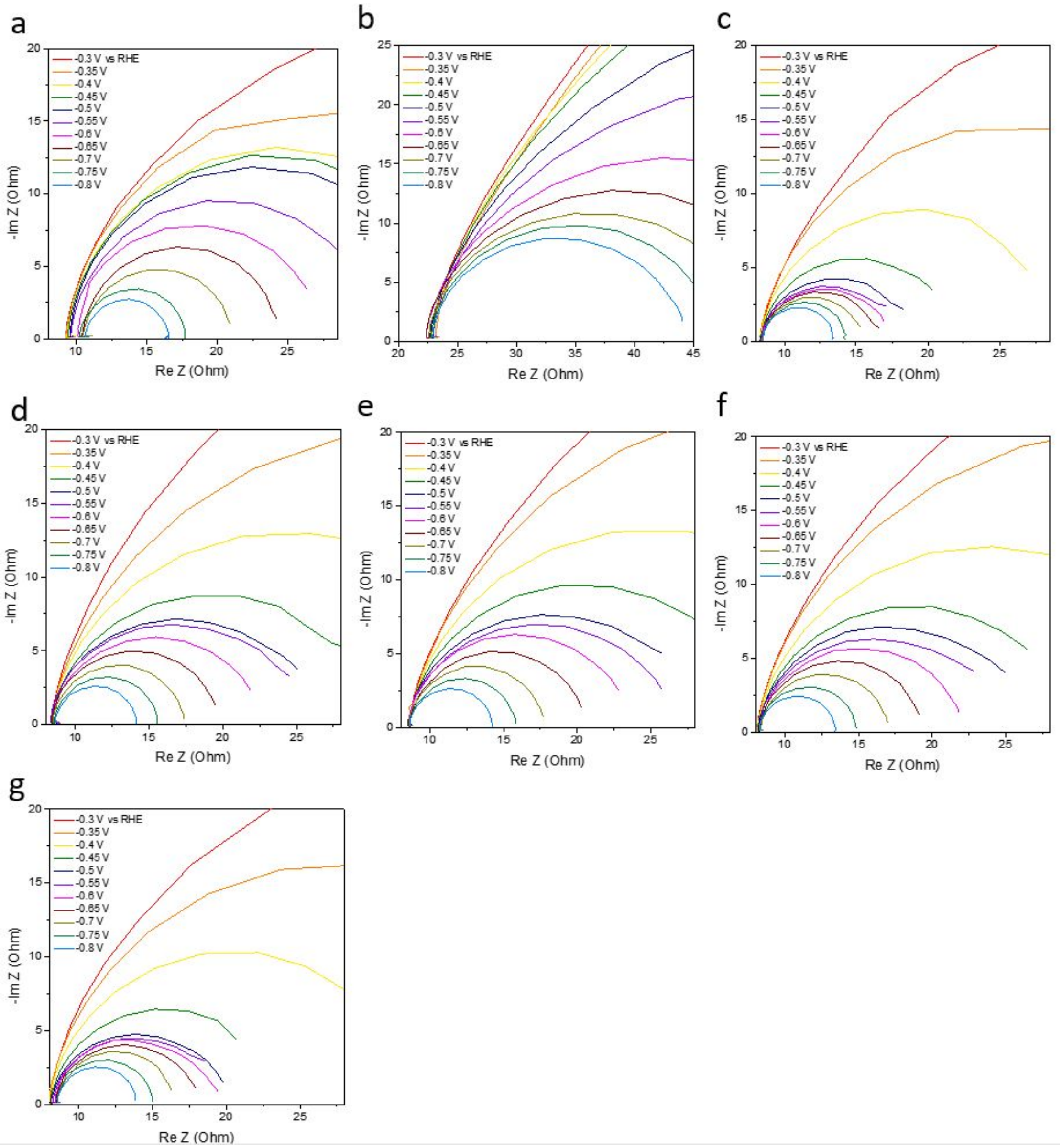

Figure S16. Nyquist plots of (a) benchmark metallic copper, (b) Cu-MOF, (c) Cu-Carbon-400C, (d) Cu-Carbon-500C, (e) Cu-Carbon-600C, (f) Cu-Carbon-700C and (g) Cu-Carbon-800C.

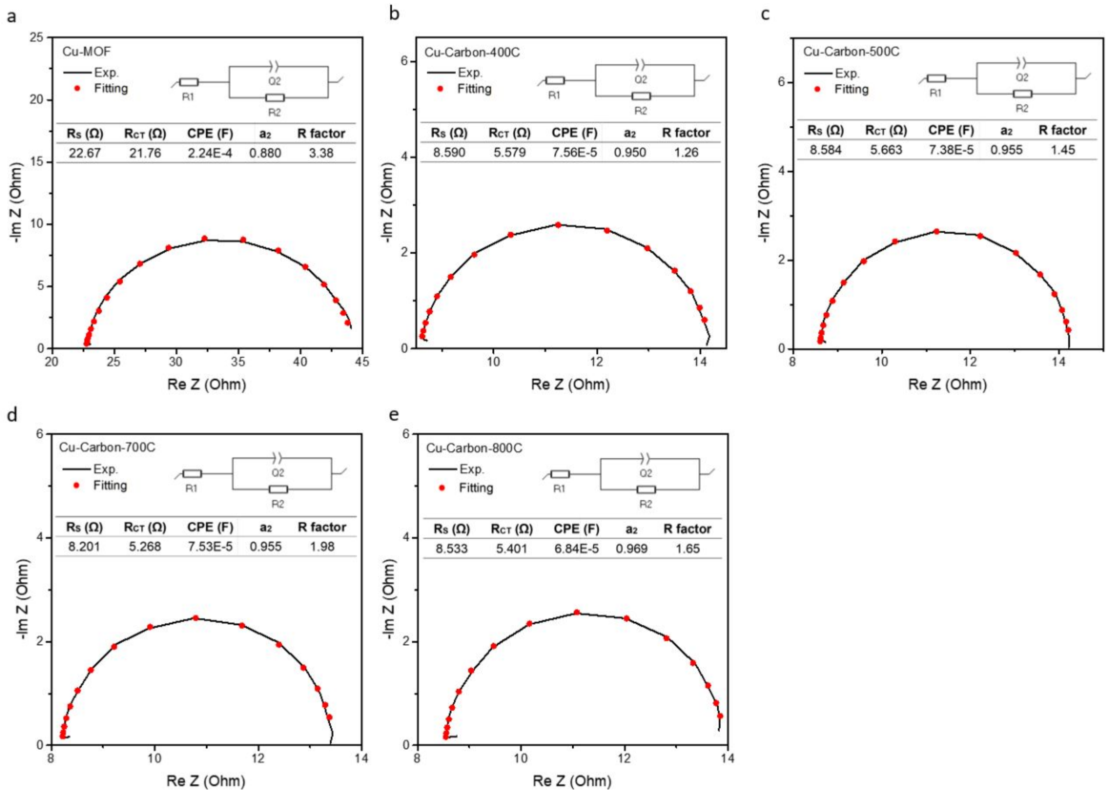

Figure S17. Equivalent circuit for fitting the Nyquist plot and Nyquist plot of (a) Cu-MOF, (b) Cu-Carbon-400C, (c) Cu-Carbon-500C, (d) Cu-Carbon-700C and (e) Cu-Carbon-800C.

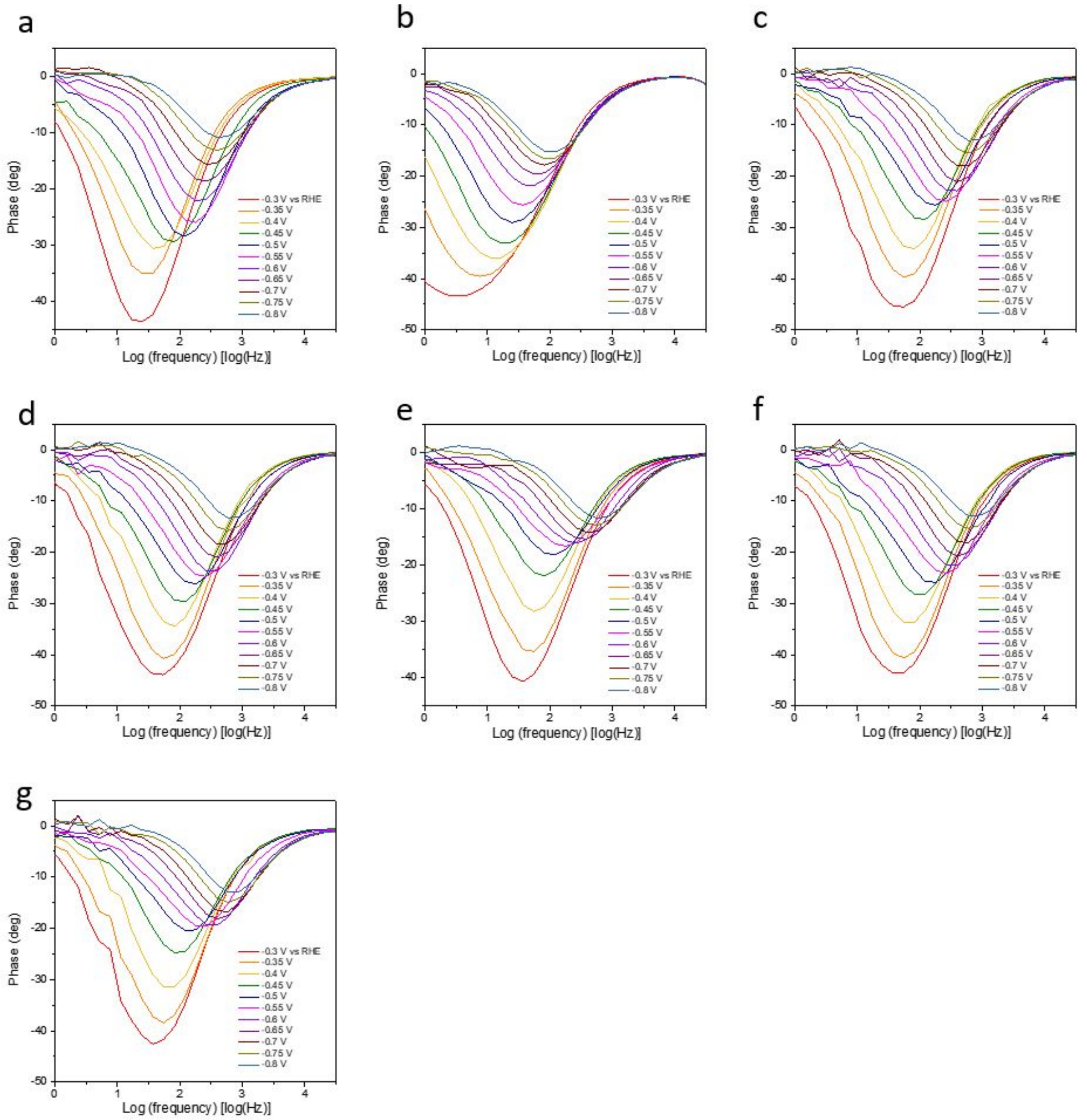

Figure S18. Bode plots of (a) benchmark metallic copper, (b) Cu-MOF, (c) Cu-Carbon-400C, (d) Cu-Carbon-500C, (e) Cu-Carbon-600C, (f) Cu-Carbon-700C and (g) Cu-Carbon-800C.

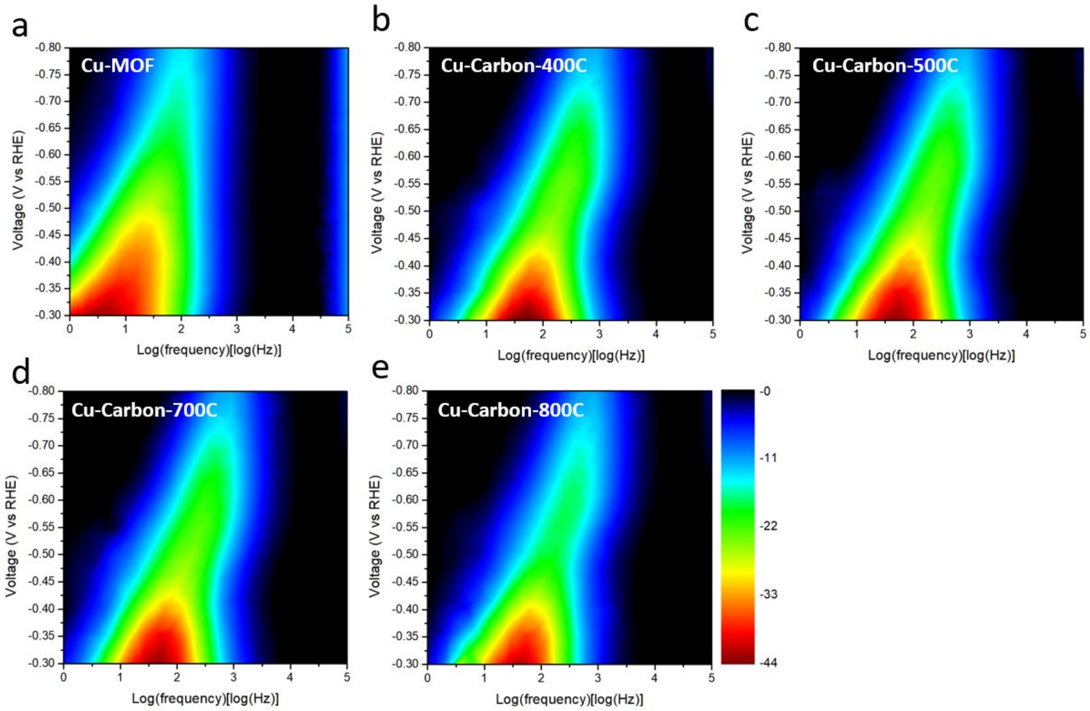

Figure S19. 2D-Bode plots of (a) Cu-MOF, (b) Cu-Carbon-400C, (c) Cu-Carbon-500C, (d) Cu-Carbon-700C and (e) Cu-Carbon-800C.

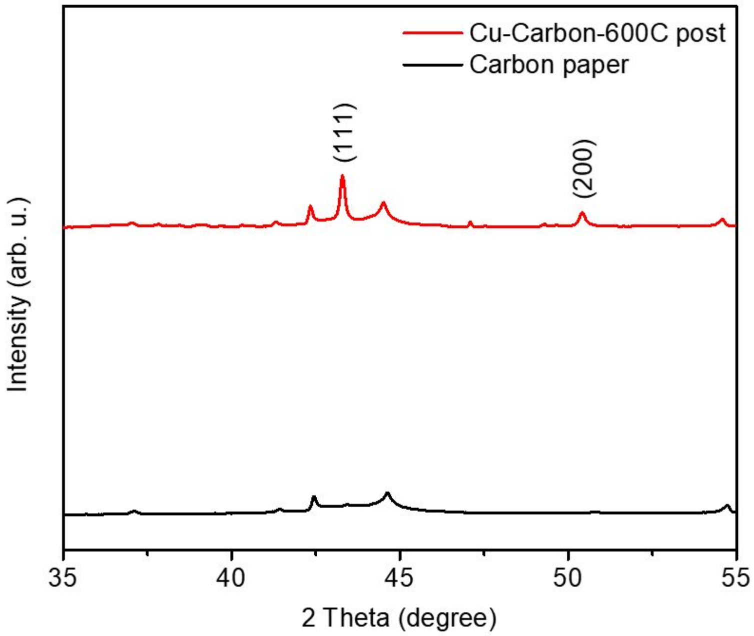

Figure S20. Post-reaction XRD of Cu-Carbon-600C and XRD of carbon paper.

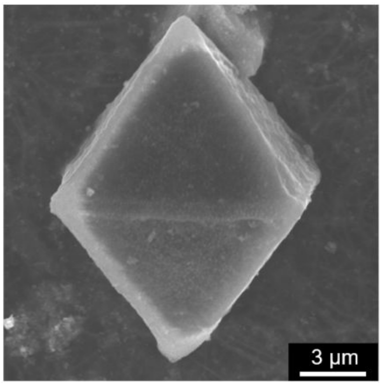

Figure S21. Post-reaction SEM of Cu-Carbon-600C.

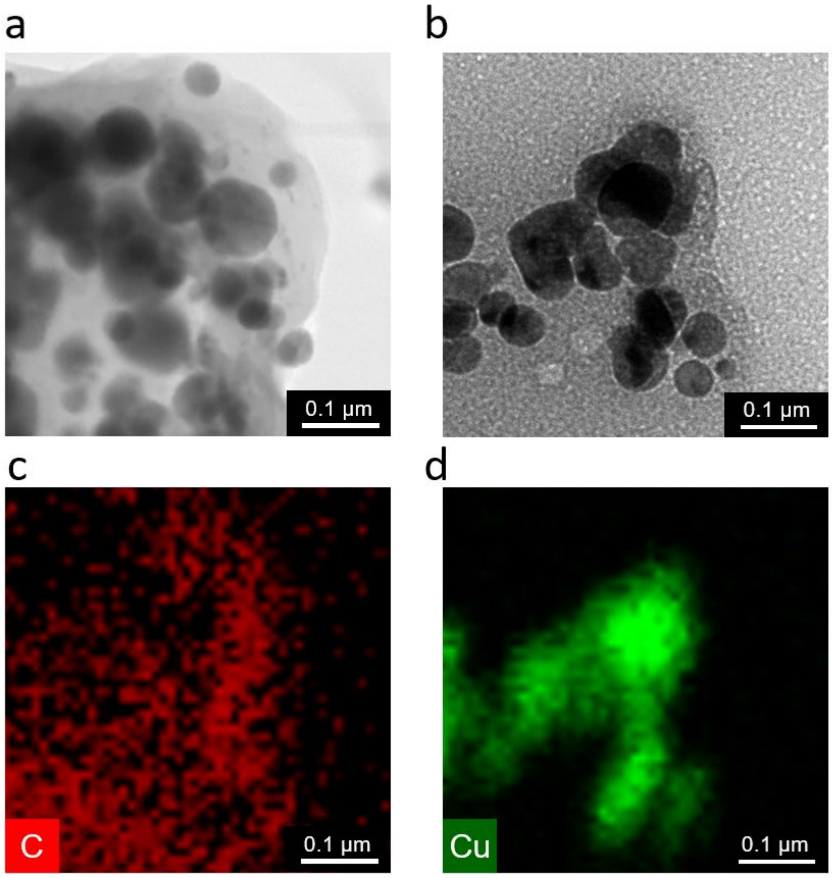

Figure S22. (a) Post-reaction TEM image of Cu-Carbon-600C. Post-reaction TEM image of Cu-Carbon-600C (b) bright field and EDX mapping of (c) C element and (d) Cu element.

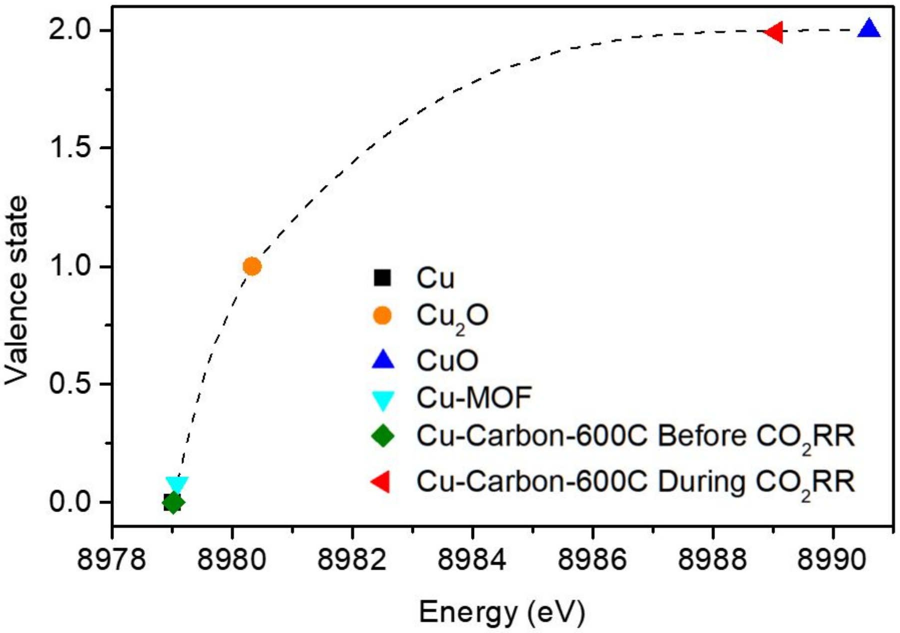

Figure S23. Valence state fitting of Cu-MOF, Cu-Carbon-600C before and during CO_2RR.

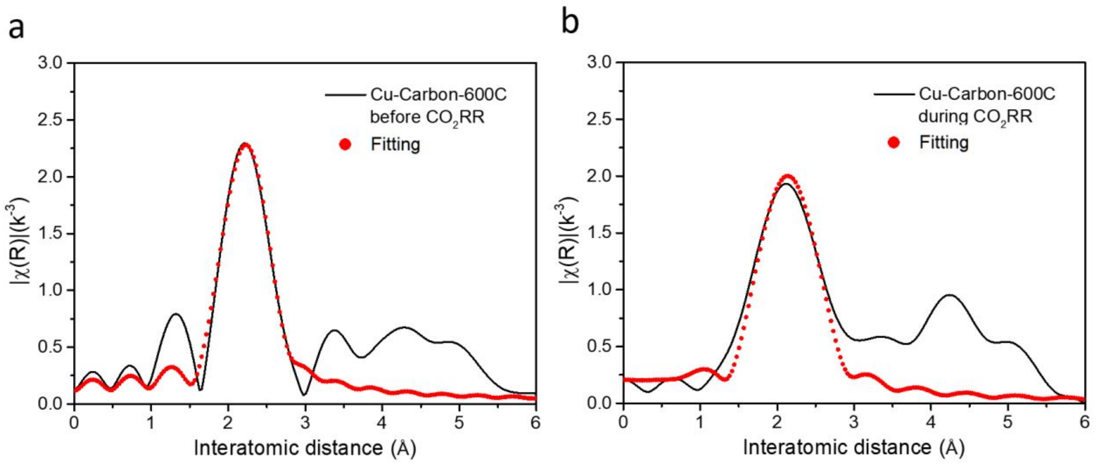

Figure S24. EXAFS fitting of Cu-Carbon-600C (a) before CO_2RR and (b) during CO_2RR.

Table S1. BET surface area of Cu-MOF and Cu-Carbon-600C

Sample	BET surface area (m^2/g)
Cu-MOF	1234
Cu-Carbon-600C	270

Table S2. Copper weight percentage of Cu-Carbon-400C, Cu-Carbon-500C, Cu-Carbon-600C, Cu-Carbon-700C and Cu-Carbon-800C.

Sample	Element: Cu wt %
Cu-Carbon-400C	57.66
Cu-Carbon-500C	58.25
Cu-Carbon-600C	60.46
Cu-Carbon-700C	62.26
Cu-Carbon-800C	69.11

Table S3. Faradaic efficiencies of CO_2RR products at various current densities for benchmark metallic copper, Cu-MOF, and Cu-Carbon-600C. N.A.

Sample	J (mA cm^{-2})	FE_{C2H4} (%)	FE_{CH4} (%)	FE_{H2} (%)	FE_{HCOOH} (%)	FE_{C2H5OH} (%)	FE_{C3H7OH} (%)
Benchmark metallic Cu	200	44.39	N.A.	6.55	8.31	11.64	N.A.
	300	50.09	N.A.	5.12	5.52	14.45	2.51
	400	47.67	4.45	6.2	4.73	15.74	N.A.
	500	41.76	8.15	9.43	4.39	16.73	N.A.
	600	33.26	14.18	15.21	4.42	15.51	N.A.
Cu-MOF	200	28.54	N.A.	11.14	9.83	9.97	N.A.
	300	36.03	N.A.	8.74	3.24	7.31	N.A.
	400	39.19	N.A.	8.67	5.59	12.80	N.A.
	500	36.13	N.A.	9.13	3.33	11.63	N.A.
	600	24.67	0.20	11.42	7.32	13.45	N.A.
Cu-Carbon-600C	200	36.64	N.A.	5.76	8.30	11.63	3.09
	300	45.9	N.A.	4.67	5.35	12.16	2.06
	400	51.24	N.A.	3.90	4.85	13.22	1.55
	500	55.48	N.A.	4.62	3.81	20.10	1.24
	600	53.11	N.A.	5.61	3.55	18.51	1.03

Table S4. Comparison of electrochemical CO_2-to-C_2 products performance.

Catalyst	FE_{C2} (%)	J_{C2} (mA/cm^2)	J_{total} (mA/cm^2)	V (vs. RHE)	References
**Cu-Graphene-600**	**75.6**	**377.9**	**500**	**-0.7**	**This work**
PdCu-BM	52.5	141.8	270	-1.7	*Adv. Energy Mater. 2024, 2402237*
c-CuO	71	241.4	340	-1.5	*Angew. Chem. Int. Ed.* 2024, e202413832
Cu_8	58.5	152.1	260	-1.3	*Angew. Chem. Int. Ed.* 2024, e202412144
Cu/BaOx	71	284	400	-0.75	*Nat. Catal.* 2022, 5, 1081
Cu(OH)BTA	72	360	500	-0.87	*Nat. Commun.* 2023, 14, 474
OD-Cu-III	72	216	300	-1.05	*J. Am. Chem. Soc.* 2022, 144, 259

Table S5. EXAFS fitting of Cu-Carbon-600C before and during CO_2RR.

	Cu-Carbon-600C Before CO_2RR	Cu-Carbon-600C After CO_2RR
Coordination number	4.8	4.7
R(Å)	2.54	2.52
σ^2	0.003	0.009
E_0(eV)	5.06	2.02
